# Target identification of mouse stem cell probe CDy1 as ALDH2 and Abcb1b through live-cell affinity-matrix and ABC CRISPRa library†

**DOI:** 10.1039/d1cb00147g

**Published:** 2021-08-20

**Authors:** Naoki Miyamoto, Young-Hyun Go, Larissa Miasiro Ciaramicoli, Haw-Young Kwon, Heon Seok Kim, Xuezhi Bi, Young Hyun Yu, Beomsue Kim, Hyung-Ho Ha, Nam-Young Kang, Seong-Wook Yun, Jin-Soo Kim, Hyuk-Jin Cha, Young-Tae Chang

**Affiliations:** Center for Self-assembly and Complexity, Institute for Basic Science (IBS) Pohang 37673 Republic of Korea ytchang@postech.ac.kr; Department of Life Science, Sogang University 35 Baekbeom-ro Mapo-gu Seoul 04107 South Korea; Department of Chemistry, Pohang University of Science and Technology (POSTECH) Pohang Gyeongbuk 37673 Republic of Korea; Division of Oncology, Department of Medicine, Stanford University School of Medicine Stanford CA USA; Bioprocessing Technology Institute, Agency for Science, Technology and Research (A*STAR) Singapore 138668 Singapore; College of Pharmacy, Sunchon National University Jungangro 255 Sunchon 57922 South Korea; Neural Circuit Research Group, Korea Brain Research Institute (KBRI) Daegu 41068 Republic of Korea; Department of Convergence IT Engineering, Pohang University of Science and Technology Pohang Gyeongbuk 37673 Korea; Nonclinical Drug Safety, Boehringer Ingelheim Pharma GmbH & Co. KG 88397 Biberach an der Riss Germany; Department of Chemistry, Seoul National University Seoul 08826 Republic of Korea; Center for Genome Engineering, Institute for Basic Science (IBS) Daejeon 34126 Republic of Korea; College of Pharmacy, Seoul National University Seoul 08826 Republic of Korea hjcha93@snu.ac.kr

## Abstract

CDy1 is a powerful tool to distingusih embryonic stem cells for reprogramming studies and regeneration medicine. However, the stem cell selectivity mechanism of CDy1 has not been fully understood. Here, we report ALDH2 and ABCB1 as the molecular targets of CDy1, elucidated by live-cell affinity-matrix and ABC transporter CRISPRa library screening. The two unique orthogonal mechanisms provide the potential of multi-demensional cellular distinction of specific cell types.

## Abstract 2

CDy1 is a powerful tool to distingusih embryonic stem cells for reprogramming studies and regeneration medicine.

## Graphical abstract

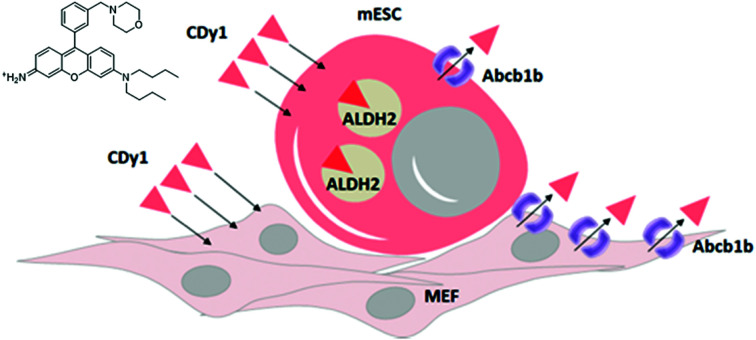

Fluorescent probes are attractive and versatile tools for live cell distinction, overcoming the limits of the antibody-based approach.^1^ We have developed a series of Diversity Oriented Fluorescence Libraries (DOFL) and successfully discovered bioimaging probes for various cell types.^2^ Pluripotent Stem cells (PSCs) are capable of self-renewing and differentiating into all types of cells in a body and of great interest as a valuable resource for regenerative medicine and developmental biology research. CDy1 (Compound of Designation yellow 1, *λ*_ex_/*λ*_em_ = 535/570 nm; Fig. 1A) was one of the powerful probes, selective towards embryonic stem cells (ESCs) and induced pluripotent stem cell (iPSC),^3^ and has been used widely in reprogramming studies and regeneration medicine.^4^ In spite of the powerful applicability, the pluripotent stem cell selectivity mechanism of CDy1 has not been fully understood. Here, for the first time, we report the molecular mechanism of CDy1 by identifying its binding and also gating target proteins.

**Fig. 1:**
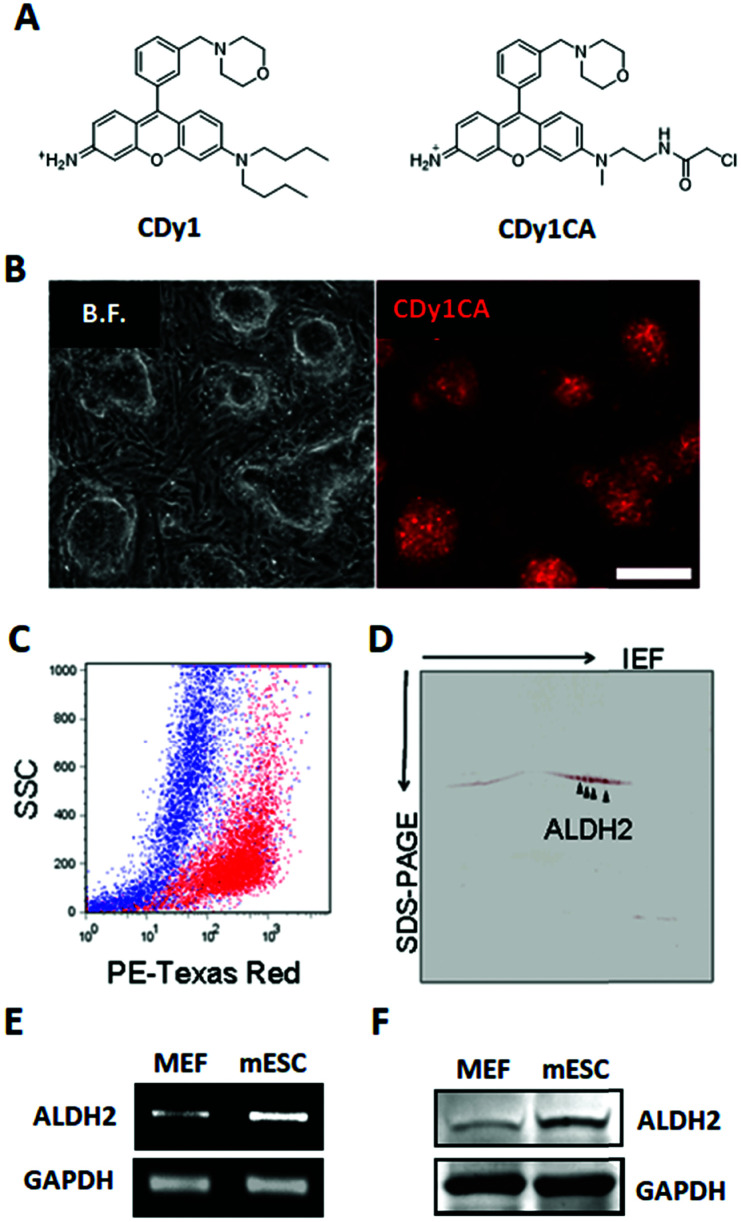
CDy1 binding protein identification. (A) Chemical structure of CDy1 (left) and CDy1CA (right). (B) Fluorescence microscopy image of CDy1CA stained mESC, scale bar, 200 μm. (C) Overlaid dot plot flow cytometry image of the CDy1CA stained mESC (red) and MEF (blue) confirmed selective staining of mESC by CDy1CA. (D) 2D gel fluorescent image of CDy1CA binding protein. Mass spectrometry analysis identified the stained spots as ALDH2. (E) RT-PCR of ALDH2 in MEF and mESC. (F) Western blot of ALDH2 in MEF and mESC.

To facilitate the identification of binding target for CDy1, we utilized a CDy1 derivative, CDy1CA, with a chloroacetyl moiety (Fig. 1A). The chloroacetyl group can form a covalent bond between the probe and a reactive thiol group of the target protein. Since the covalent labeling can be carried out in live cells and the target protein can be easily visualized through the fluorescent property of the probe, the chloroacetyl moiety has been successfully used for the molecular target identification.^5^ In the previous report, CDy1CA also exhibited selectivity towards neuronal stem cells over differentiated cells in neurosphere, and has been used for the monitoring of symmetric/asymmetric stem cell division.^6^ As expected, CDy1CA selectively stains mouse ESC (mESC) in a similar manner to its mother compound, CDy1 confirmed by fluorescence image (Fig. 1B) and flow cytometry analysis (Fig. 1C). After incubating mESC with CDy1CA, the proteins extracted from the mESC were subjected to 2D gel electrophoresis and imaged using the fluorescence signal. The gel image showed specifically stained four spots, which were excised, trypsinized and analyzed by MALDI-TOF/TOF tandem mass spectrometry, giving aldehyde dehydrogenase 2 (ALDH2) as the binding protein of CDy1CA (Fig. 1D and Fig S1, ESI†).

To confirm the specific binding of CDy1CA to ALDH2, a competition assay with CDy1 was performed. The preincubation of mESC with CDy1 inhibited the interaction between CDy1CA and its binding protein, ALDH2, in a dose dependent manner (Fig. S2, ESI†). Genomic mRNA expression analysis by DNA microarray showed that ALDH2 expression is much higher in mESC compared to that in mouse embryonic fibroblast (MEF), a typical differentiated counterpart of mESCs (Fig. S3, ESI†). The gene expression trend was further confirmed by RT-PCR and western blotting of ALDH2 in mESC and MEF (Fig. 1E and F) ALDH2 was reported to localize in mitochondria for a crucial role in aldehyde detoxification^7^ and well matches with the mitochondrial localization of CDy1. The relevance of ALDH2 as the cell selectivity origin was further confirmed by the knockdown experiment of ALDH2 by shRNA. The efficient knockdown of ALDH2 in mESC was confirmed by RT-PCR and western blotting (Fig. 2A), and fluorescence intensity in mESC reduced with the decrease in the ALDH2 expression level (Fig. 2B and C). The binding of CDy1CA with ALDH2 was also inhibited by an ALDH inhibitor, disulfiram, in a dose dependent manner (Fig. S4, ESI†). These results demonstrate that CDy1 selectively binds to ALDH2 as its binding target inside mESC, and the high expression of ALDH2 in the embryonic stem cell is responsible for the cell selectivity of CDy1.

**Fig. 2:**
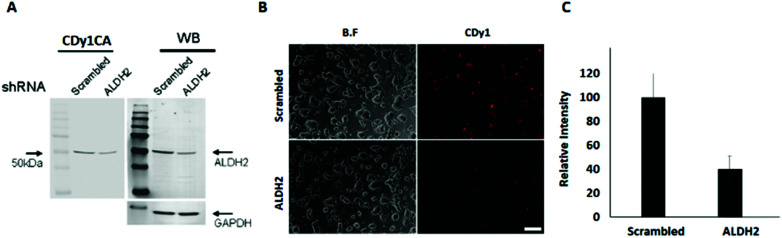
ALDH2 knockdown in mESC and CDy1 staining change. (A) ALDH2 knockdown using shRNA. Left panel: CDy1CA signal from the ALDH2 shRNA transfected mESC extract compared to scrambled shRNA. Right panel: Western blotting of ALDH2 by shRNA transfection. (B) Fluorescence image of mESC with CDy1 after ALDH2 knockdown, scale bar, 200 μm. (C) Relative fluorescence intensity of CDy1 in mESC after ALDH2 knockdown.

Interestingly, there was a report that drug resistant multiple myeloma cells were selectively isolated by CDy1 as a dim population of staining, and the molecular target was identified as ABCB1, one of the ATP-binding cassette (ABC) transporters.^8^ To evaluate the possible role of transporters in CDy1 selectivity, we checked the CDy1 staining pattern on mESC and MEF under fixed or unfixed condition (Fig. S5, ESI†). The fixed cells showed almost the same degree of the CDy1 signal, but unfixed MEF showed a decrease in the CDy1 intensity compared with mESC, implying the contribution of active transporters for the cell selectivity. Then, we hypothesized that transporters on the plasma membrane may also have an essential role for the kinetic flow of CDy1 in mESC and MEF.^9,10^

There are hundreds of transporters for molecules to be imported or exported, and we mainly focused on ABC transporters, which generally work as efflux gate proteins.^11^ We constructed CRISPR activation (CRISPRa) library^12,13^ targeting 153 types of transporters including all 48 human ABC transporters^8,14^ for the systematic screening of the specific ABC transporters to efflux CDy1 from the cells. Up to ten sgRNAs were designed to the promoter region of each transporter gene as the sgRNA library (Table S1, ESI†), and the sgRNA library was transduced into HeLa cells expressing catalytically dead Cas9 (dCas9) fused with VP64-p65-Rta (VPR; transcriptional activator) by the lentiviral infection method.^12^ In this way, we successfully generated the ABC-CRISPRa library pool, overexpressing a transporter in every single cell, so CDy1 can be exported from the cell when the target transporter is overexpressed. We sorted 10% of CDy1^dim^ population after staining the library pool with CDy1 in every selection round to enrich the cell population exporting CDy1 (Fig. 3A and B). In round 2, CDy1^dim^ population clearly appeared compared with that of the library pool. Also, round 3-sorted cells showed strong enrichment of CDy1^dim^ population (Fig. 3B). The fluorescence between library cells and round 3-sorted cells were clearly separated and not overlapped at all. The CDy1 signal of round 3-sorted cells is similar to the intensity of unstained cells.

**Fig. 3:**
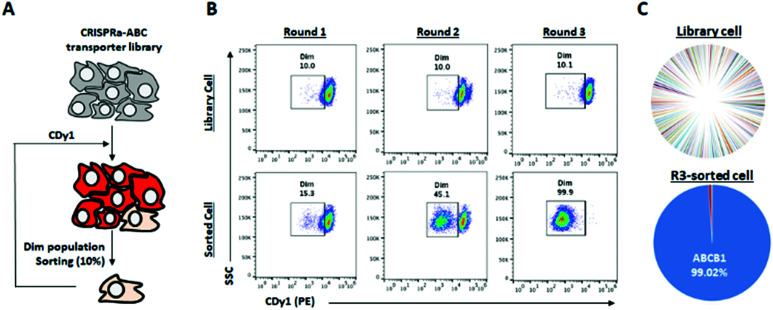
Target ABC transporter identification for CDy1 with CRISPRa library of ABC transporters. (A) Schematic view of target ABC transporter bio-panning with CRISPRa-ABC transporter library (153 transporters, 10 sgRNAs/transporter). Then, CRISPRa-ABC transporter library was generated. This library cells were stained with CDy1. (B) Cells were stained with 100 nM CDy1 for 30 min, and 10% dim population was collected by cell sorter and subjected for next round of selection. (C) The distribution of sgRNAs in library cells and round 3-sorted cells, analyzed by NGS.

Next, we extracted the genomic DNA from the library pool and round 3-sorted cells, and their sgRNA distribution was analyzed by next generation sequencing (NGS). The NGS result showed that 99% of sgRNAs were targeting ABCB1 in round 3-sorted cells, which is the same target with the drug resistant multiple myeloma (Fig. 3C). It demonstrates that ABCB1 is the specific efflux transporter for CDy1; thereby, the gene expression level in mESC is supposed to be lower than in MEF. We examined the mRNA expression level of Abcb1a and Abcb1b, which are mouse homologous of human ABCB1 in mESC and MEF (Fig. S6, ESI†). While Abcb1a was not detected in both cells, MEF showed about 2-fold higher Abcb1b expression than in mESC. From these results, we postulated that the efflux transporter Abcb1b may also have an essential role for CDy1 to stain mESC stronger than MEF.

To confirm that Abcb1b is a suitable efflux transporter for CDy1, we tested CDy1 staining on MEF with known ABCB1 inhibitors. Under higher concentration of ABCB1 inhibitors, verapamil and cyclosporine A, the CDy1 signal was stronger in the treated MEF compared to that of the control cells by fluorescence images (Fig. 4A) and flow cytometry analysis (Fig. 4B, Fig. S7 and S8, ESI†). We also conducted the Abcb1b siRNA knockdown experiment on MEF for further validation. MEF was treated with three kinds of Abcb1 siRNAs, generating various knockdown effects (Fig. 4C). The CDy1 staining signal of each clone was compared with that of the negative control cells. The lower the expression level of Abcb1b, the stronger the CDy1 staining signal was with a good quantitative correlation (Fig. 4D and Fig. S9, ESI†). Based on these inhibitors and knockdown experiment results, we conclude that Abcb1b is responsible for CDy1 efflux and contributes the cell selectivity of CDy1 to mESC over MEF.

**Fig. 4:**
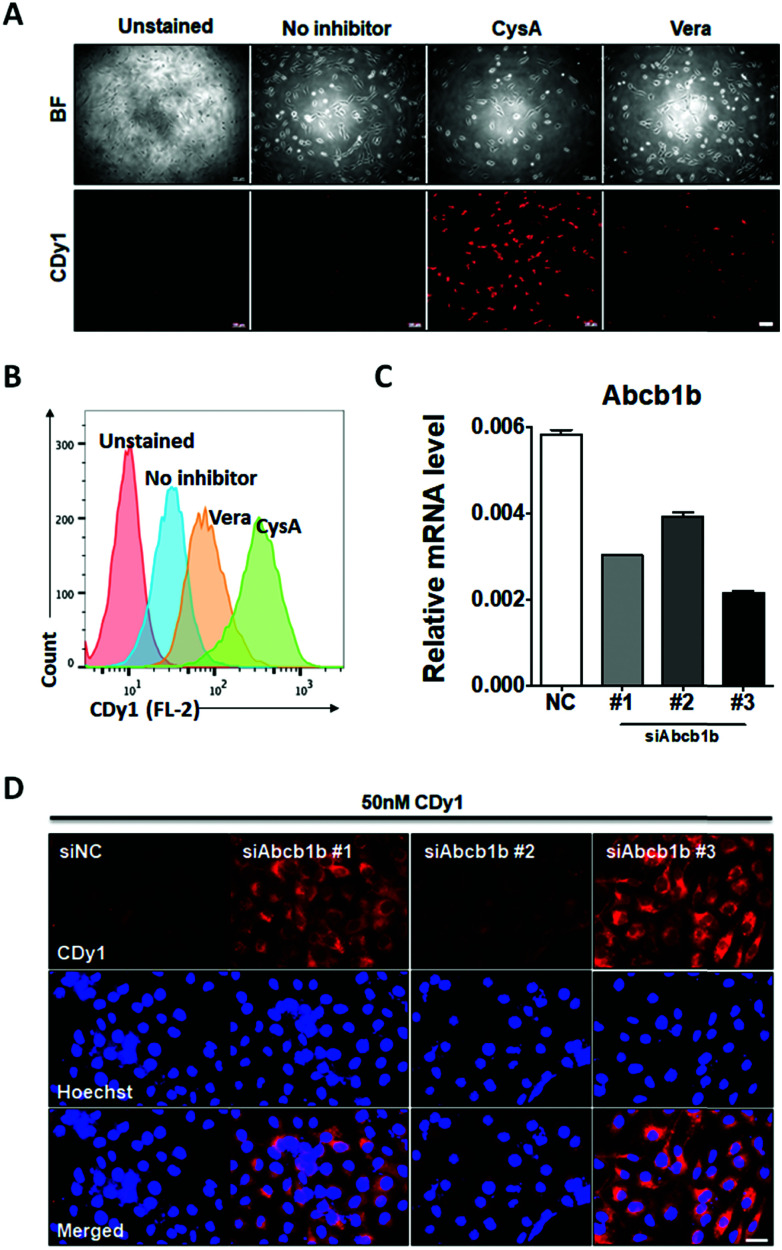
CDy1 signal in MEF treated with the ABCB1 inhibitor and siRNA. (A) Fluorescence images of CDy1 stained MEF, treated with ABCB1 inhibitors, verapamil (Vera: 50 μM) or cyclosporine A (CysA: 10 μM), scale bar 100 μm. (B) Flow cytometry data for theABCB1 inhibitor effect. (C) Abcb1b mRNA expression by MEF knockdown using negative control siRNA (siNC) and three siRNAs targeting Abcb1b (siAbcb1b #1-3) for 48 h. (D) Fluorescence images of MEF with 50 nM CDy1 after the knockdown of Abcb1b, scale bar 50 μm.

In summary, we identified ALDH2 as the binding target of CDy1 in mESC. Following the mechanism classification,^9^ this may be a case of Holding Oriented Live-cell Distinction (HOLD). In HOLD, there are mainly two subcategories of protein and carbohydrate targeting. As ALDH2 is a selectively overexpressed protein in mESC, the detailed mechanism would be defined as Protein-Oriented Live-cell Distinction (POLD). In addition, we elucidated another transporter, Abcb1b, which further enhances the selectivity of CDy1 in mESC over MEF. The transporter-based mechanism has been described as a Gating-Oriented Live-cell Distinction (GOLD).^9^ In CDy1 case, it seems both POLD and GOLD play important roles together to increase the cell staining selectivity in mESC (Fig. 5). The GOLD mechanism would determine the flow in and out balance, and POLD will increase the residing tendency of the probes inside cell or in specific organelles (mitochondria, in CDy1 case). In this report, we emphasize the possible combination of multiple mechanisms to increase the cell selectivity of the probes or drug molecules. In addition, the understanding of CDy1 staining mechanism itself could render new insights into stem cell research.

**Fig. 5:**
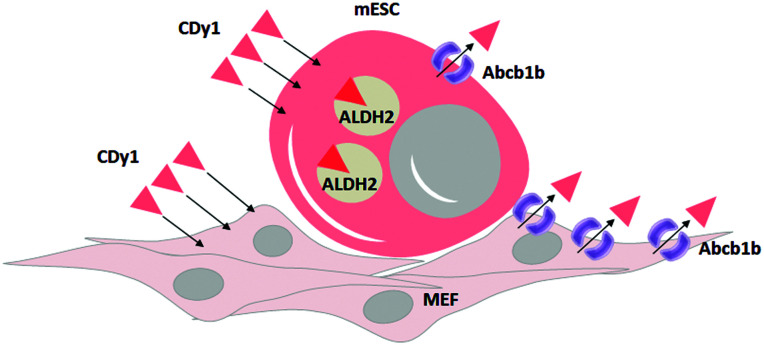
Proposed mechanism of CDy1 for mESC over MEF by protein target of ALDH2 and a negative gating target Abcb1b.

## Conflicts of interest

There are no conflicts to declare.

## Supplementary Material

CB-002-D1CB00147G-s001
