# Supplementary material for: Target identification of mouse stem cell probe CDy1 as ALDH2 and Abcb1b through live-cell affinity-matrix and ABC CRISPRa library
Source: RSC Chem Biol. 2021 Aug 20;2(6):1590–3. doi: 10.1039/d1cb00147g (PMC8637918; doi:10.1039/d1cb00147g)
Supplement: CB-002-D1CB00147G-s001 [file CB-002-D1CB00147G-s001.pdf]

Electronic Supplementary Material (ESI) for RSC Chemical Biology.
This journal is © The Royal Society of Chemistry 2021

Target identification of mouse stem cell probe CDy1 as ALDH2 and Abcb1b through live-cell affinity-matrix and ABC CRISPRa library

Naoki Miyamoto[‡a], Young-Hyun Go[‡b], Larissa Miasiro Ciaramicoli[‡c], Haw-Young Kwon[a], Heon Seok Kim[d], Xuezhi Bi[e], Young Hyun Yu[f], Beomsue Kim[g], Hyung-Ho Ha[f], Nam-Young Kang[h], Seong-Wook Yun[i], Jin-Soo Kim[j,k], Hyuk-Jin Cha*[l], and Young-Tae Chang *[a,c]

a. Center for Self-assembly and Complexity, Institute for Basic Science (IBS), Pohang 37673, Republic of Korea. E-mail: ytchang@postech.ac.kr
b. Department of Life Science, Sogang University, 35 Baekbeom-ro, Mapo-gu, Seoul 04107, South Korea
c. Department of Chemistry, Pohang University of Science and Technology (POSTECH), Pohang, Gyeongbuk 37673, Republic of Korea
d. Division of Oncology, Department of Medicine, Stanford University School of Medicine, Stanford, CA, USA
e. Bioprocessing Technology Institute, Agency for Science, Technology and Research (A*STAR), Singapore 138668, Singapore
f. College of Pharmacy, Sunchon National University, Jungangro 255, Sunchon 57922, South Korea
g. Neural Circuit Research Group, Korea Brain Research Institute (KBRI), Daegu, 41068, Republic of Korea
h. Department of Convergence IT Engineering, Pohang University of Science and Technology, Pohang, Gyeongbuk 37673, Korea
i. Nonclinical Drug Safety, Boehringer Ingelheim Pharma GmbH & Co. KG, 88397 Biberach an der Riss, Germany
j. Department of Chemistry, Seoul National University, Seoul 08826, Republic of Korea
k. Center for Genome Engineering, Institute for Basic Science (IBS), Daejeon 34126, Republic of Korea
l. College of Pharmacy, Seoul National University, Seoul, 08826, Republic of Korea

Correspondence Email: ytchang@postech.ac.kr

hjcha93@snu.ac.kr

Contents

- Experimental
- Fig. S1: MALDI-TOF/TOF spectra for CDy1CA-positive spots on 2D gel electrophoresis.
- Fig. S2: CDy1 vs. CDy1CA competition assay.
- Fig. S3: Expression of ALDH gene family in mESC and MEF.
- Fig. S4: CDy1CA binding inhibition by ALDH inhibitor disulfiram.
- Fig. S5: CDy1 staining pattern in mESC and MEF.
- Fig. S6: Abcb1b expression level.
- Fig. S7: Verapamil treatment with CDy1 staining on MEF.
- Fig. S8: Verapamil or cyclosporine A treatment with CDy1 staining on MEF.
- Fig. S9: CDy1 signal of MEF treated with siRNAs targeting Abcb1b.
- Table S1: The list of sgRNAs targeting ABC transporter genes.

Experimental

Cell Culture

mESC (J1 cell line) and MEF (NIH3T3 cell line) were purchased from the American Tissue Culture Collection (ATCC, VA, USA). mESC was cultured in a culture dish coated with 0.1% gelatin using a high-glucose Dulbecco's Modified Eagle's medium (DMEM) supplemented with 10% fetal calf serum, 2 mM L-glutamine, 100 U/ml penicillin, 100 μg/ml streptomycin, 0.1 mM non-essential amino acids, 0.1% ß–mercaptoethanol and 100 U/ml leukemia inhibitory factor (LIF, Chemicon). MEF was maintained in the same media as used for mESC without LIF and treated with mitomycin C (10 μg/ml) before used as feeder cell.

Image-based cell screening

MEFs were plated on 0.1% gelatin-coated 384-well cell culture microplates (Greiner bio-one, Germany). On the next day, mESCs were plated both on MEF feeder and feeder-free wells for overnight culture and then incubated with 500 nM of rosamine fluorescent compounds. After 0.5 h, 24 h and 48 h, TRITC fluorescence and bright field images were taken using ImageXpressMICRO imaging system (Molecular Devices).

Flow cytometry to analyze CDy1CA signal on mESC and MEF

MEFs and mESCs were cultured in 60 mm culture dish for 24 h in the presence of 500 nM of hit compounds including CDy1 or 2 μM CDy1CA. The cells were harvested by trypsin treatment, washed with PBS and resuspended in PBS. The fluorescence intensity of the cells was measured on Flow Cytometry (BDTM LSR II) with PE-Texas Red filter (excitation at 488 nm, emission at 615 nm). The dot plot images for each cell type were overlaid using FlowJo7 (Three Star Inc.).

Target protein identification by 2DE-MALDI-TOF-TOF

Protein Extraction – mESCs incubated with 2 μM of CDy1CA for 24 h were trypsinized and washed three times with cold PBS. The cell pellet was resuspended in a lysis buffer (40 mM Trizma, 7 M Urea, 2 M thiourea and 4% CHAPS (Sigma) premixed with 10 μl/ml Protease Inhibitor Cocktail (EDTA free, GE healthcare) and 50 μg/ml DNase I and 50 μg/ml RNase A (Roche). The cell extract was homogenized by vortexing followed by ultrasonication on ice for 10 second, and incubated for 30 min at room temperature. The supernatant was collected after centrifugation at 20,000 x g for 45 min at 10ºC and protein concentration was determined by Bradford protein assay reagent (Bio-Rad).

Two-dimensional gel electrophoresis (2-DE) – Isoelectric focusing (IEF) was performed using

PROTEAN IEF Cell (Bio-Rad) with 18 cm ReadyStrip pH 3-10NL (Bio-Rad). The sample of 1 mg protein was diluted into 340 µl of rehydration buffer (7 M urea, 2 M thiourea, 4% CHAPS, 20 mM DTT, 0.5% IPG buffer pH 3-10 NL (GE healthcare), and loaded to each IPG strip with passive rehydration and focused for 60,000 Vhrs at 20ºC. The IEF strips were reduced in equilibration buffer I (50 mM Tris-HCl, pH 8.8, 6 M urea, 30% glycerol, 2% SDS, 2% DTT) for 10 min and alkylated with SDS-PAGE Equilibration Buffer II (50 mM Tris-HCl, pH 8.8, 6 M urea, 30% glycerol, 2% SDS, 2.5% iodoacetamide, and a trace of bromophenol blue) for an additional 10 min. The equilibrated IEF strips were embedded in 0.5% agarose in 1x Tris-glycine-SDS buffer on top of a second dimension SDS-PAGE (12%) gel. After electrophoresis for 5 h at 30 mA, the 2D fluorescence image was acquired using the Typhoon 9400 scanner (GE healthcares) at excitation/emission wavelengths of 532 nm/580 nm with PMT at 500V. The fluorescence labeled protein spots were directly excised from the gel. After spot cutting, the gel rescanned again confirming the labeled protein were accurately removed and stained with 0.1% Coomassie brilliant blue R250 in 50% methanol, 10% acetic acid in water.

Trypsin digestion and MALDI-TOF/TOF MS and MS/MS analyses – Fluorescent spots excised from the gel were washed with Mili-Q water and cut into 1 mm^3 in size, then incubated with 200 µl of 50% acetonitrile/25 mM ammonium bicarbonate buffer, pH7.8 for 3 times and dehydrated in 100% acetonitrile. The gel pieces were rehydrated with 10 ng/µL trypsin gold, Mass Spectrometry grade (Promega) in 25 mM ammonium bicarbonate buffer, pH 8.0, overlaid with 10 µl of 25 mM ammonium bicarbonate buffer, and digested for 16 h at 37ºC. The peptides were extracted sequentially with 20 mM ammonium bicarbonate buffer followed by 50% acetonitrile in 0.1% TFA. The pooled peptides were speed-vac dried and dissolved in 4 µl 0.1% TFA. Tryptic peptide (0.5 µL) was spotted onto Prespotted AnchorChip target plate (Bruker Daltonics) according to manufacturer's protocol. The peptide mass fingerprint and lift MS/MS of selected precursors were acquired in UltraFlex III TOF-TOF (Bruker Daltonics) with the compass 1.2 software package including flexControl 3.0 and flexAnalysis 3.0 with PAC peptide calibration standards. The peak list was submitted to in-house Mascot server 2.2 (http://phenyx.bii.a-star.edu.sg/search_form_select.html) through BioTools 3.1 (Bruker Daltonics) with the database of IPI-mouse containing 56692 sequences with peptide mass tolerance of 100 ppm with maximum 1 missed cleavage, considering of variable modifications of carbamidomethyl at cysteine (C) and Oxidation at methionine (M).

ALDH2 knock down using shRNA

mESC was directly infected with $1x10^5$ infectious units of virus (IFU) of shRNA lentiviral particles prepared in complete medium containing 10 µg/mL of Polybrene. shRNA was carried out in suspension with scrambled control or ALDH2 shRNA lentiviral particles (Santa Cruz Biotechnology) in a 6-well cell culture plate. The infected mESCs were seeded at a density of 1.5 x 10^5/well on a gelatin-

coated 6-well plate. On the next day, the culture medium was replaced with complete medium and the cells were incubated for overnight before infected cell selection using Puromycin dihydrochloride at a concentration 10 ug/mL. The cells were split 1:3 and the medium with Puromycin was replaced every 3-4days until resistant colonies were identified.

Western blotting

Protein samples prepared by same method as described in the section Target protein identification by 2DE-MALDI-TOF-TOF were subjected to an SDS-PAGE and transferred to PVDF membrane (Bio-Rad). The membranes were incubated with PBS containing 5% (w/v) nonfat dry milk and 0.05% Tween-20 for 1 h at room temperature and then K15 goat-anti-human ALDH2 (dilution 1:500, Santa Cruz Biotechnology) was added for overnight incubation at 4ºC. The antibody-antigen complex was detected by incubation with donkey anti-goat IgG-PE-Cy5 secondary antibody (Santa Cruz Biotechnology) prepared in PBS containing 1% BSA, and the membrane was scanned using Typhoon 9400 scanner (GE healthcare).

CDy1 vs CDy1CA competition assay

Free compound, CDy1 was pre-incubated with mESCs at concentrations of 0, 5, 15 and 30 μM for 1 h and 2 μM of CDy1CA was added for 30 min incubation. Total cell lysates were prepared in a lysis buffer described in the section Target protein identification by 2DE-MALDI-TOF-TOF. Equal amounts of protein from each sample were subjected to an SDS-PAGE. The fluorescent signal of CDy1CA was visualized using Typhoon 9400 scanner (GE healthcare).

DNA microarray analysis

Total RNA was isolated using RNeasy Mini Kit (QIAGEN Inc.) from MEF and mESC. Each sample was converted to biotinylated cRNA using Illumina RNA Amplification Kit (Ambion Inc.) according to the manufacturer's instructions. The cRNA was purified and hybridized to the MouseRef-8 v2.0 Expression BeadChip. Arrays were scanned with iScan Scanner System (Illumina Inc.). Data analyzed by using GenePlex v 3.0 software (ISTECH Inc.). Using GenePlex Preprocessing module, global median normalization procedures were applied, and genes with ''present'' flag value in all samples were used for DEG (Differentially expressed gene) finding analysis.

RT-PCR for ALDH2 and ALDH1a1 on mESC and MEF

Total RNA was isolated using RNeasy Mini Kit (QIAGEN) from cells according to the manufacturer's instruction. cDNA was synthesized from 1 μg of total RNA using RevertAid™ H Minus First strand cDNA Synthesis kit (Fermentas) and amplified by PCR using GoTaq® Green Master Mix (Promega). The PCR condition was as follows: 30 s denaturation at 95°C, 30 s annealing at 60°C, and a final 30 s

of extension at 72°C. The sequences of primers used in this study are: ALDH1a1 (access no. NM_013467.3): sense 5'-GCACTCAATGGTGGGAAAGT-3', antisense 5'-CCATGGTGTGCAAACTCAAC-3'; ALDH2 (access no. NM_009656.3): sense: 5'-AGGCGAGGACTTGTTCTTCA-3', antisense: 5'-CGGAGACATTTCAGGACCAT-3'; GAPDH (access no. XM_001473443.1): sense 5'-GCACAGTCAAGGCCGAGAAT-3', antisense 5'-GCCTTCTCCATGGTGGTGAA-3' and Nanog (access no. NM_028016.1): sense 5'-AGGGTCTGCTACTGAGATGCTCTG-3', antisense 5'-CAACCACTGGTTTTTCTGCCACCG-3'. The PCR products obtained after 25 cycles were resolved on 1% agarose gels, visualize by ethidium bromide staining and the images were taken using Gel Logic 200 Imaging System (Kodak).

ALDH2 knock down using siRNA

mESC was directly transfected with 30 nM of siRNA duplexes prepared in 7.5 μL of Lipofectamine RNAiMAX reagent (Invitrogen) according to the manufacturer's instructions. siRNA transfection was carried out in suspension with scrambled control and ALDH2 siRNA (Santa Cruz Biotechnology) duplex in a 6-well cell culture plate. The transfected mESCs were seeded at a density of 1.5 x 105/well on a gelatin-coated 6-well plate. The cells were collected 72 h later.

Disulfiram inhibition of CDy1CA binding

Disulfiram was pre-incubated with mESCs at concentrations of 0, 3, 10, 30 and 50 μM for 1 h and 2 μM of CDy1CA was added for 30 min incubation. The samples were processed for visualization as described in the section CDy1 vs CDy1CA competition assay.

ABC-CRISPR activation Screen

The construction protocol of CRISPRa library cell for transporters was described before.[7] $1.5x10^6$ of the library cells were cultured in 10 cm culture plate for 3 days. Then, the cells were incubated in DMEM including 100 nM CDy1 for 30 min. After washing the cells with PBS for three times, cells were detached with trypsin. Collected cells were analyzed and their live single cells were sorted with gating of 10% dim population in fluorescence intensity (FL2) on S3e Cell Sorter (BioRad). Sorted dim population was cultured and expanded for next round. Analysis of CDy1 signal on sorted cells (staining condition was same with above) was performed on Moflo XDP (Beckman coulter) with PE-filter. The FCS data was analyzed on FlowJo 10.4.2 software.

NGS analysis of integrated sgRNAs in enriched cells

Genomic DNA was isolated from library cells and round-3 enriched cells with QIAamp® DNA Mini Kit (QIAGEN) according to the manufacturer's protocol. NGS protocol was described before.[7]

RT-PCR for Abcb1b on mESC and MEF

Abcb1 and Sox2 mRNA expression levels on mESC and MEF were analyzed with each primers (mAbcb1b forward: 5'-ATGGATCCCAGAGTGACACT-3', mAbcb1b reverse: 5'-CTTTCTGTGGACACTTCTG-3', mSox2 forward: 5'-ATGGGCTCTGTGGTCAAGTC-3', mSox2 reverse: 5'-CCCTCCCAATTCCCTTGTAT-3) on RT-PCR procedures described above.

Flow cytometry to analyze CDy1 staining pattern on mESC and MEF

mESC and MEF were fixed under 4% PFA condition for 10 min. Then, fixed and unfixed cells were incubated with 50 nM CDy1 for 1 h. After washing cells with PBS for three times, CDy1 signal was analyzed on flow cytometry with FL-2 filter.

Inhibitors study against Abcb1b on mESC and MEF

mESC and MEF were cultured under verapamil (50, 25, 12.5, 6.25 or 3.125 μM) or cyclosporine A (10, 5, 2.5 or 1.25 μM) for overnight (DMSO was utilized as no inhibitor condition.). Then, these cells were incubated with 50 nM CDy1 for 1 h. After washing cells with PBS for three times, CDy1 signal was analyzed on flow cytometry with FL-2 filter or imaged on microscope.

Abcb1b knock down using siRNA

MEF were transfected with 20 μM of siRNAs with Dharmafact for 48 h. After transfection, these cells were incubated with 50 nM CDy1 for 1 h. The cells were washed with PBS for three times, and then CDy1 signal was analyzed on flow cytometry with FL-2 filter or imaged on microscope. Abcb1b mRNA expression level was analyzed on the same procedure with above.

Supplementary Figures

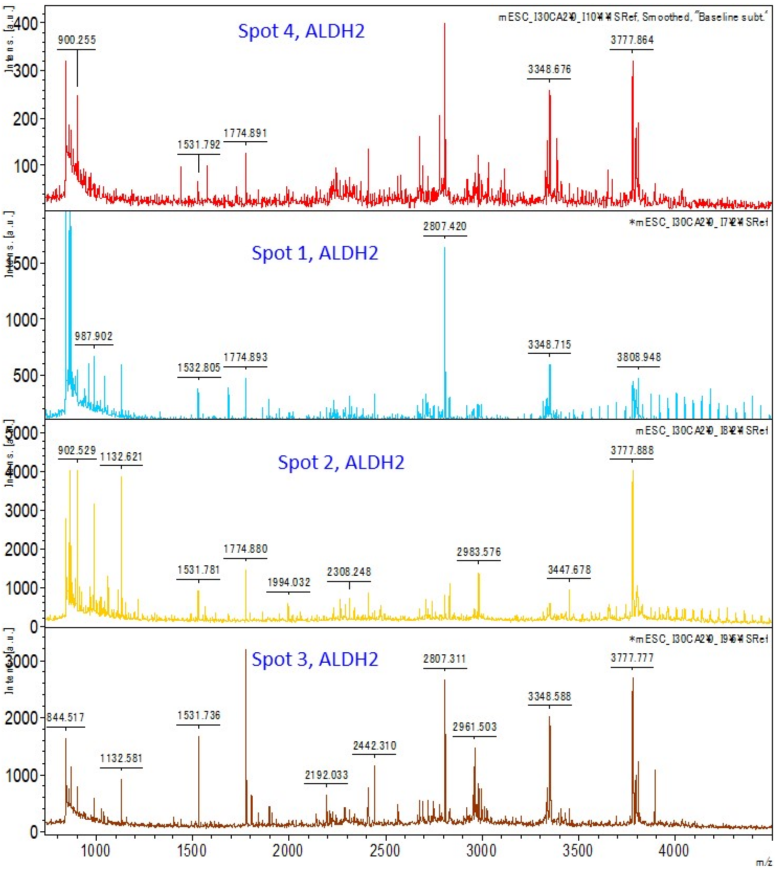

Fig. S1 MALDI-TOF MS spectra of trypsin digested peptides for CDy1CA-positive spots on 2D gel electrophoresis. Each spots are shown in Fig. 1D. Horizontal axis is m/z value and vertical axis is the intensity of peptide ions. All of the four protein spots were identified as ALDH2 by mascot database search using tryptic peptide mass fingerprint and selected precursor LIFT MS/MS fragment ions.

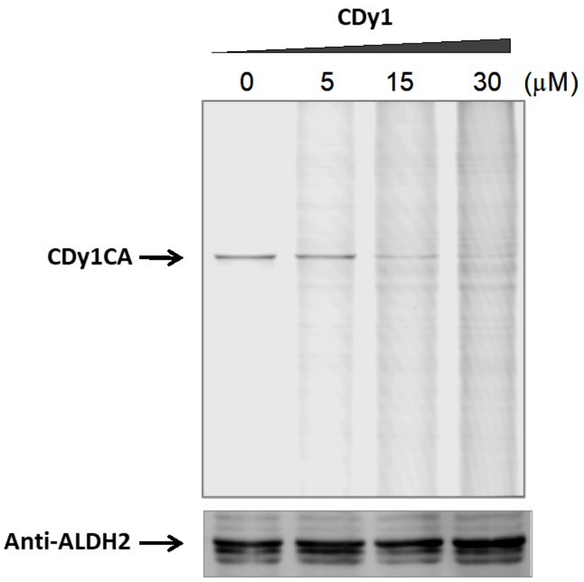

Fig. S2 CDy1 vs. CDy1CA competition assay. Upper panel: fluorescent SDS-PAGE gel image showed CDy1CA binding to its target protein was inhibited by CDy1. Lower panel: Western blotting showed ALDH2 levels in the protein samples were similar.

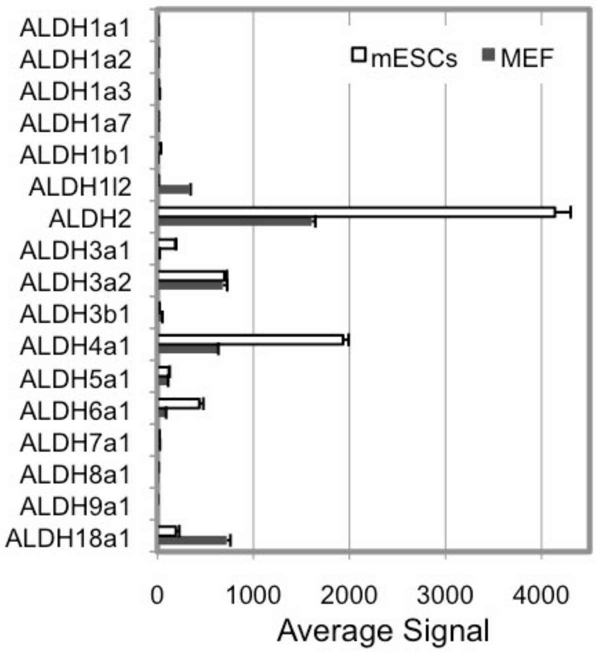

Fig. S3 Expression of ALDH gene family in mESC and MEF. Higher expression of ALDH2 in mESC than in MEF was demonstrated by DNA microarray

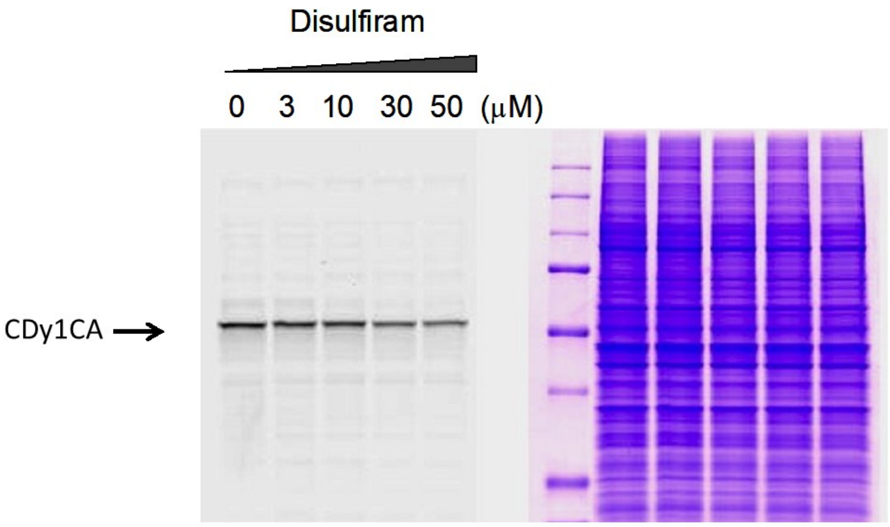

Fig. S4 CDy1CA binding inhibition by ALDH inhibitor disulfiram. The cells were preincubated with different concentrations of disulfiram followed by CDy1CA staining. Left panel: the SDS-PAGE shows CDy1CA binding to its target protein in inhibited by disulfiram in concentration dependent manner, right panel: the same gel was stained with Coommassie brilliant blue to assess the amount of loaded protein.

.

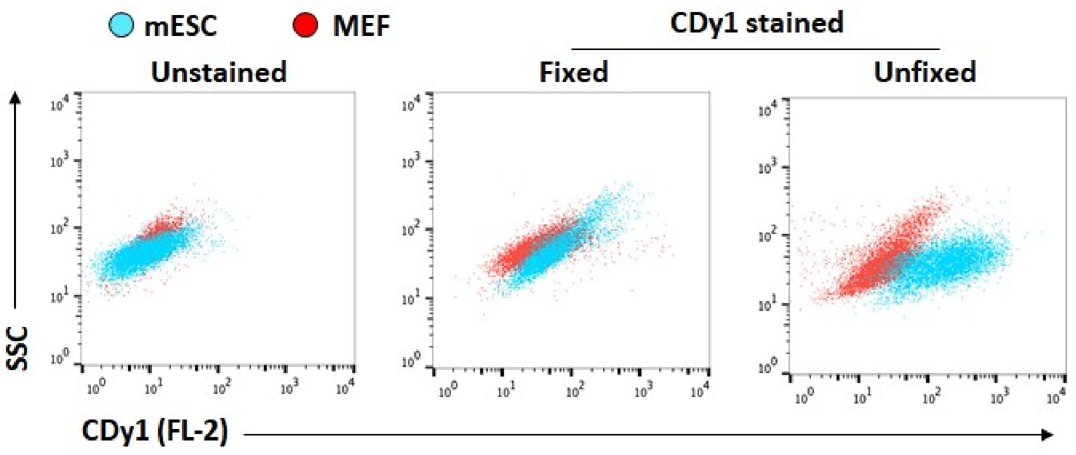

Fig. S5 CDy1 staining pattern in mESC and MEF. CDy1 staining pattern on mESC and MEF was tested in fixed or unfixed condition. In fixed condition, both cells showed similar CDy1 signal compared with unstained condition's. However, MEF, expressing Abcb1b more highly than mESC, showed less CDy1 signal than mESC at unfixed condition (live cell condition).

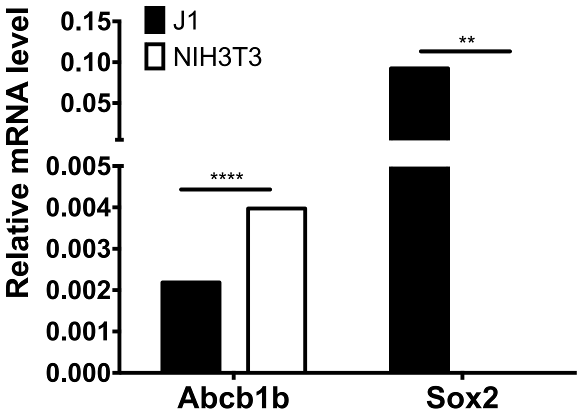

Fig. S6 Abcb1b expression level. mRNAs expression level of Abcb1b and Sox2, stem cell marker, were compared in mESC and MEF.

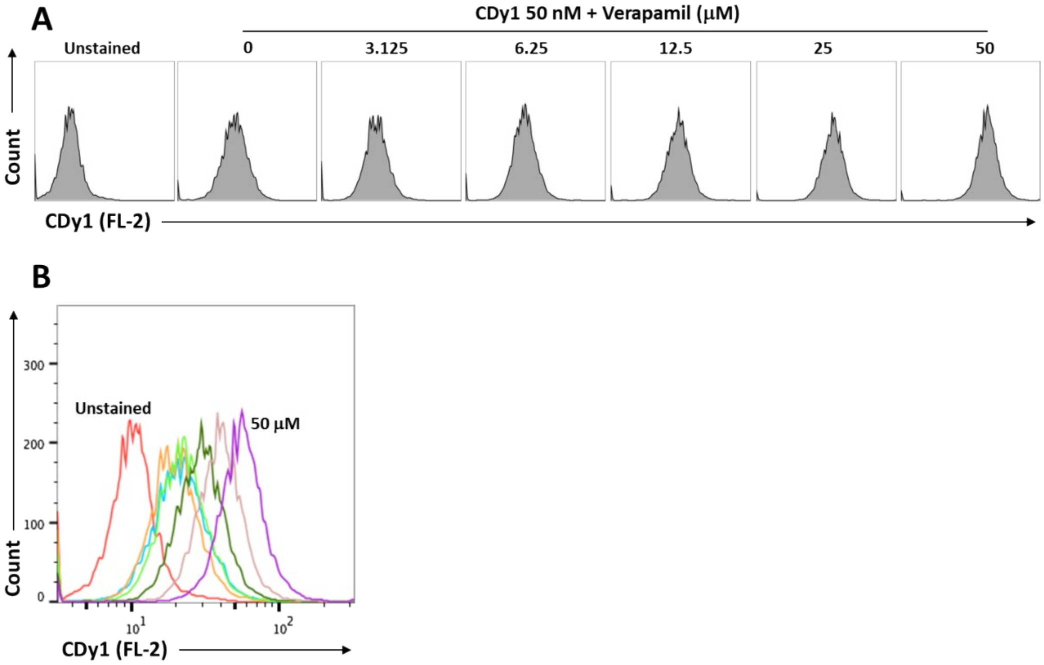

Fig. S7 Verapamil treatment with CDy1 staining on MEF. (A) MEF was incubated with DMSO or verapamil, an ABCB1 inhibitor, at 3.125, 6.25, 12.5, 25 or 50 μM for 16 hours. After staining cells with 50 nM CDy1 for 1 hour, and washing them with PBS, CDy1 intensity was analyzed on FACS. Dependent on verapamil concentration, CDy1 signal was increased. (B) Overwriting histograms of (A). Red histogram means unstained cells, cyan histogram means 50 nM CDy1 without verapamail condition, orange histogram means 50 nM CDy1 with 3.125 μM verapamail condition, light green histogram means 50 nM CDy1 with 6.25 μM verapamail condition, dark green histogram means 50 nM CDy1 with 12.5 μM verapamail condition, light purple histogram means 50 nM CDy1 with 25 μM verapamail condition and dark purple histogram means 50 nM CDy1 with 50 μM verapamail.

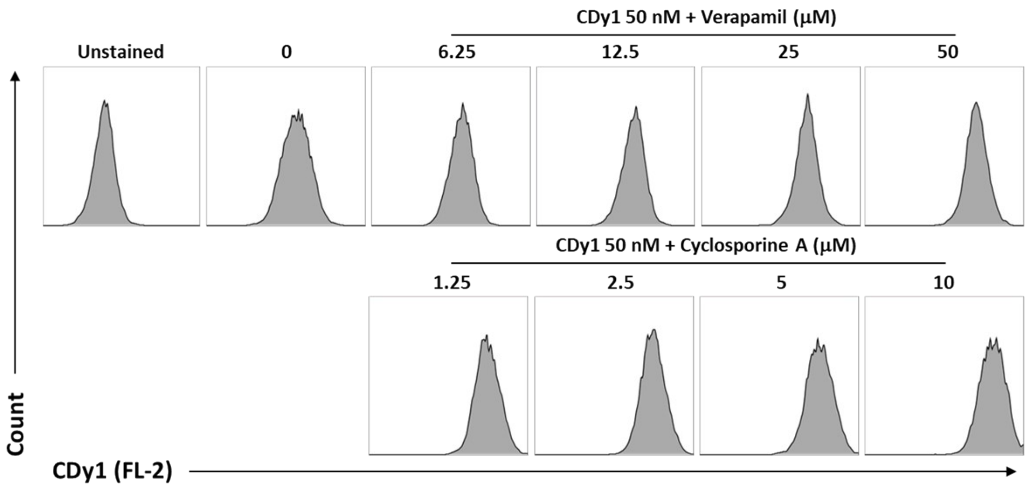

Fig. S8 Verapamil or cyclosporine A treatment with CDy1 staining on MEF. MEF was treated with verapamil or cyclosporine A for 24 hours. Then, the cells were stained with 50 nM CDy1 for 1 hour. After washing the cells with PBS, CDy1 signals were analyzed on flow cytometry.

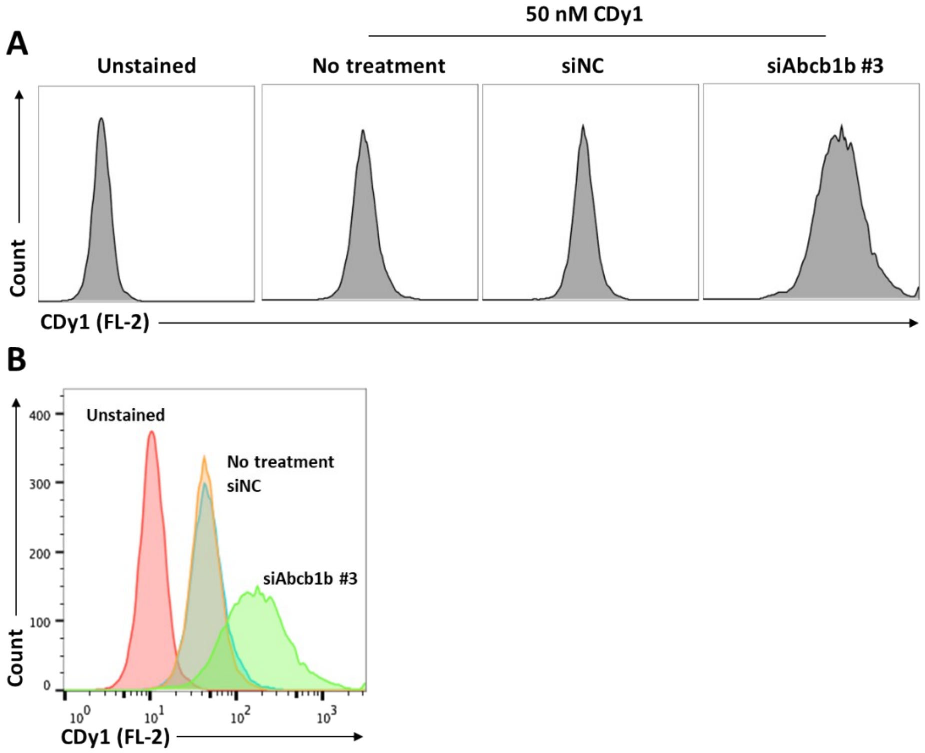

Fig. S9 CDy1 signal of MEF treated with siRNAs targeting Abcb1b. (A) MEF was treated with 20 nM control siRNA (siNC) or three kinds of siRNAs targeting Abcb1b (siAbcb1b) for 48 hours. Then, cells were stained with 50 nM CDy1. After washing cells with PBS, CDy1 signal was detected on flow cytometry. (B) Overwriting data of (A). Red histogram means unstained cells, orange histogram means cells untreated with siRNA, cyan histogram means siNC-treated cells and light green means siAbcb1b-treated cells.

Table S1. The list of sgRNAs targeting ABC transporter genes

Gene	Target sequence	Count in library cell	Count in round 3-sorted cell
ABCA1	GACTCTCTAGTCCACGTTCC	67	0
ABCA1	GCAATTACGGGGTTTTTGCC	197	0
ABCA1	CCGATAGTAACCTCTGCGCT	80	0
ABCA1	CTCCCTAGATGTGTCGTGGG	1	0
ABCA1	AACGTCGCCCGTTTAAGGGG	29	0
ABCA1	GGCTGAACGTCGCCCGTTTA	16	0
ABCA1	GCTGAACGTCGCCCGTTTAA	139	0
ABCA1	GGTGCAGCCGAATCTATAAA	51	0
ABCA1	AGCAGAAAGCACGTGGAGCC	68	0
ABCA1	CTGAACGTCGCCCGTTTAAG	102	0
ABCA2	CCACATCGAATCCGAGACCC	29	0
ABCA2	CGGGGTCTCGGATTCGATGT	0	0
ABCA2	GCTCTTCTCCCAGAGGCATC	82	0
ABCA2	CGAAGGCTAAGCCGGGGTCT	21	0
ABCA2	GACCCCGGCTTAGCCTTCGT	42	0
ABCA2	GGCTTAGCCTTCGTAGGCCG	92	0
ABCA2	CCGGCTTAGCCTTCGTAGGC	64	0
ABCA2	GGAGCGCGGCGCCTTTAAGC	0	0
ABCA2	CGGCCTACGAAGGCTAAGCC	0	0
ABCA2	GCGCTGCGCTCTTCTCCCAG	344	0
ABCA3	CACACCCGGAAACTCGACTT	69	0
ABCA3	ACAGAGCAGTGCGCAAGCAC	0	0
ABCA3	ACGGCCAAAGTCGAGTTTCC	74	0
ABCA3	GCAGTGGAAGTCTTCACACC	27	0
ABCA3	CGCAGAGTCATTCCTGTCAG	33	0
ABCA3	AACGGCCAAAGTCGAGTTTC	25	0
ABCA3	CACAGAGCAGTGCGCAAGCA	79	0
ABCA3	TGCCCGCGCCTATGTCCAGC	6	0
ABCA3	GCCCGCGCCTATGTCCAGCA	208	0
ABCA3	GGTGCGAACCGCGTACTCGC	0	0
ABCA4	GTCCTTCTCCTGGTGATTAA	174	0
ABCA4	CTCAGGAACCCCTCTCATGG	0	0
ABCA4	GATCTGCCGAGTGAGTCGAT	38	0

ABCA4	GCCAGTCAAACCCTGACTGC	87	0
ABCA4	GGCACTCTCCTCCATGAGAG	39	0
ABCA4	TTCCTGAGGGGGATGTCTTT	2	0
ABCA4	GCACCAGGGAAATTGAGGTG	71	0
ABCA4	AATGGGAAACCAAGACCCTC	20	0
ABCA4	AGCCTTTAATCACCAGGAGA	277	0
ABCA4	TCCATGAGAGGGGTTCCTGA	104	0
ABCA5	CACGCTCTTTCTGGAGGCAC	184	0
ABCA5	GGATCTCGCCACGCTCTTTC	8	295
ABCA5	CCACGCTCTTTCTGGAGGCA	80	0
ABCA5	TCTCGCCACGCTCTTTCTGG	46	0
ABCA5	GGAGGCACGGGCAAAAACCA	52	0
ABCA5	CCTCCTGCAGAGCGCACCTG	58	0
ABCA5	TCTAGTGGCTCAGGGGCCCT	0	0
ABCA5	AGGCCCGACTCTAGTGGCTC	89	0
ABCA5	TGCAGAGCGCACCTGAGGGC	79	0
ABCA5	GGCCTCGTGGCTCTTCCCCA	268	0
ABCA6	GGAAATGACTGTTCCGTGGT	31	0
ABCA6	TACACCCCTTTTCAGAAACC	98	0
ABCA6	TCATCATGTACAGCTTGGTC	42	0
ABCA6	ACACAGCCTGGTTTCTGAAA	181	0
ABCA6	TGACTGTTCCGTGGTTGGTT	97	0
ABCA6	GCCTTTCAACTACAGGGATA	87	0
ABCA6	GCAAGGAAATGACTGTTCCG	0	0
ABCA6	ATGACTGTTCCGTGGTTGGT	35	0
ABCA6	AAGGGGGAAAACGGTAAACA	63	0
ABCA6	TCTTGTTCCTGCCTCCATCT	210	0
ABCA7	ACGAGTGTAGTCCCAGAGTC	15	0
ABCA7	GGTTTCTTAAAGGGACCGCG	47	0
ABCA7	GGAGGAGACGGGATCACAGA	0	0
ABCA7	GGCTCTTGCTGCATCACCGT	78	0
ABCA7	GCCGGTTATACAACGTGGGG	0	0
ABCA7	GAACGAGGCTGCGCTGTCTT	0	0
ABCA7	GGACTAAACTAGAGGGAGCC	1	0
ABCA7	CAGAGGGAACTCCTGCAATT	48	0
ABCA7	ACTCCTGCAATTCGGAGCTG	127	0

ABCA7	TTGCAGCCGGTTATACAACG	0	0
ABCA8	GGCATTTATACTGGGTTGCC	82	0
ABCA8	CACATTCACGTCATCATGCT	51	0
ABCA8	CGTGAATGTGATGTTTGGCA	120	0
ABCA8	ACGTGAATGTGATGTTTGGC	28	0
ABCA8	GAAGACTGCTTTCAGAGTGG	63	0
ABCA8	TATAAATGCCACTGCTCTGC	20	0
ABCA8	CAGAGCAGTGGCATTTATAC	92	0
ABCA8	TGCCACTGCTCTGCAGGAAC	74	0
ABCA8	TGTGAAGACTGCTTTCAGAG	141	0
ABCA8	AAAATGAAGCTGGGAAGCAG	47	0
ABCA9	GAACTGAGACCAGATTCTCC	112	0
ABCA9	TTCCTGCTAGTTACTCAGCA	214	0
ABCA9	TGTAGTTGCAGATGGTTGGC	140	0
ABCA9	CTCCCTGCTGAGTAACTAGC	24	0
ABCA9	AGGGAGTTTGTAGTTGCAGA	11	0
ABCA9	TTTCCTGCTAGTTACTCAGC	120	0
ABCA9	TGCTCTACTCTTCTCTGGAT	270	0
ABCA9	TGGGGTCAACTCTCCCATTA	210	0
ABCA9	TTGGGGTCAACTCTCCCATT	107	0
ABCA9	GAGAAAGGCTAGATTCTCCT	45	0
ABCA10	TTACCTTTCCCCTCTCTCAT	38	0
ABCA10	TCAGTGTGATAATACGGGGG	40	0
ABCA10	CAATCTCTCCTATGAGAGAG	0	0
ABCA10	TAACAAAGTGCAGGCTGAGT	152	0
ABCA10	TCTCCTATGAGAGAGGGGAA	39	0
ABCA10	ATAACAAAGTGCAGGCTGAG	81	0
ABCA10	CTCAATCTCTCCTATGAGAG	39	0
ABCA10	AATACGGGGGAGGAATTAAT	63	0
ABCA10	ATAGCACAATAACAAAGTGC	0	0
ABCA10	TCAATCTCTCCTATGAGAGA	43	0
ABCA12	TCATGGAGAGTAGGAGGTGT	42	0
ABCA12	GGACCCAGATCAGTATCTTT	191	0
ABCA12	CTGGATGCGTCACAGGGACT	162	0
ABCA12	TGGATGCGTCACAGGGACTA	67	0
ABCA12	GCCACCCAAAGATACTGATC	30	0

ABCA12	AGGACCCAGATCAGTATCTT	123	0
ABCA12	GATGCGTCACAGGGACTAGG	78	0
ABCA12	AAGTCCTACTGGCCTTGCAA	183	0
ABCA12	TTCATGGAGAGTAGGAGGTG	89	0
ABCA12	ACCTGCACTGGATGCGTCAC	131	0
ABCA13	TAAGCACCATCCTTACAGGC	68	0
ABCA13	GTTCTGTGGGTTCTCGGAAA	70	0
ABCA13	ATGCCCGAGAAAGTCCCTGA	188	0
ABCA13	GTCGCACAGATCAGTTCTGT	197	0
ABCA13	TAGCTGTTCAGTGTTTCAGG	30	0
ABCA13	TGTGGGTTCTCGGAAAGGGA	36	0
ABCA13	TCAGCAGATGCCGAACAAGA	147	0
ABCA13	AGGATGGTGCTTATATCTGC	0	0
ABCA13	CTGTGGGTTCTCGGAAAGGG	0	0
ABCA13	CACTAGCTGTTCAGTGTTTC	42	0
ABCB1	AACCCAAGGATAAGTTTGGG	0	2042
ABCB1	CCTCCACCCAAACTTATCCT	165	0
ABCB1	CTGGCTAAACTTCTCAACTC	61	6559
ABCB1	CTAAGCCATGTAACTCTTCG	168	24726
ABCB1	GTAGCTCCTCCTCTGGTACT	89	0
ABCB1	TGTAGCTCCTCCTCTGGTAC	190	8777
ABCB1	GAGTTACATGGCTTAGGGAT	7	51124
ABCB1	GTTACATGGCTTAGGGATTG	115	34722
ABCB1	AGGAGGAGCTACATGAACTA	154	64849
ABCB1	AGTGTTTATCCCAGTACCAG	0	234
ABCB4	TCTCAGGAGGTCAAAAACAG	9	0
ABCB4	TGAAACAAGAGTGGGAGATA	29	0
ABCB4	GAAACAAGAGTGGGAGATAA	123	0
ABCB4	AAACAAGAGTGGGAGATAAG	194	0
ABCB4	GCAGAAATATGAAACAAGAG	0	0
ABCB4	TCTCAATGGTATTCTACAAT	75	0
ABCB4	ACTTTCTTTAAAATAGGAGG	33	0
ABCB4	GATAAGGGGACTCAGCTCTC	57	0
ABCB4	CTAGTGACTTTAATTCTCAA	142	0
ABCB4	CAGAAATATGAAACAAGAGT	66	0
ABCB5	ACAGCCATGGATATACTGGA	0	0

ABCB5	GTAGGTAGGTAAAGAGGACA	137	0
ABCB5	GGATATACTGGAAGGACTTG	34	0
ABCB5	AAGGCGGTAGGTAGGTAAAG	41	0
ABCB5	AATGAAAGAGACAAAGCCAT	179	0
ABCB5	AGAGGTATTTTTAAGGCGGT	36	0
ABCB5	GTATTTTTAAGGCGGTAGGT	0	0
ABCB5	GGCTGTAATGTCAAATAATC	168	0
ABCB5	CATTACAGCCATGGATATAC	118	0
ABCB5	GAGAAGTTATGAGACAGAAT	41	0
ABCB6	TCTCGCGCGGCTAGAATCCT	0	0
ABCB6	GTCGTCAGATTTCCCGCCCT	156	0
ABCB6	CCTCGGAGACATTTGGCCAA	125	0
ABCB6	ACCTCCACGGAACTGTGGGT	331	0
ABCB6	ATTGGCCAAATGTCTCCGAG	328	0
ABCB6	CCATTGGCCAAATGTCTCCG	144	0
ABCB6	ATTTGGCCAATGGGAGCCGT	71	0
ABCB6	GCGCGAGACAACAAGAAGTC	51	0
ABCB6	TTCCGTGGAGGTTTCCAGCT	29	0
ABCB6	AATGGGATGGGGACTCTGCC	112	0
ABCB7	GGACACTGGCGGACTGAATT	111	0
ABCB7	ACAATCAAAGCTCGGCCCTA	64	0
ABCB7	CCAATATTCCCGGTTGTGTA	120	0
ABCB7	AGGGGAGCGTGACATTGAGT	42	0
ABCB7	CTCCTCTTCTTGTTTTCCCA	226	0
ABCB7	CCTTACACAACCGGGAATAT	31	0
ABCB7	AGAGGATTATGGGACACTGG	61	0
ABCB7	CAGAATCCATTTCCGGTCTG	32	0
ABCB7	GGAGCGTGACATTGAGTTGG	69	0
ABCB7	GCGTGACATTGAGTTGGAGG	22	0
ABCB8	GCTTTTGCCAGGTGCTCCTG	58	0
ABCB8	CTACACGCCCTTGAGACGAC	156	0
ABCB8	TAGAACGCGGGTTTTGGGGG	22	0
ABCB8	TGGATACTCCATAGCTTGGA	130	0
ABCB8	CTAGATGTCCCGTCGTCTCA	287	0
ABCB8	GGCGTGTAGAACGCGGGTTT	0	0
ABCB8	GACGGGACATCTAGAGATCC	22	0

ABCB8	GCGTGTAGAACGCGGGTTTT	122	0
ABCB8	TCTACACGCCCTTGAGACGA	0	0
ABCB8	CTCAAGGGCGTGTAGAACGC	43	0
ABCB9	ATGTGGGAGGAGTTTGCCAC	24	0
ABCB9	GAAACCATTTCCGTCCCCTC	111	0
ABCB9	TTGAGACAGGCTGATGGACG	26	0
ABCB9	TAACTGGCCTTTTTCCCTGG	105	0
ABCB9	CCATTTCCGTCCCCTCAGGA	109	0
ABCB9	TGGCAAACTCCTCCCACATC	135	0
ABCB9	AAGGAGGCCAGCTTTTTAAC	142	0
ABCB9	ACGGAAATGGTTTCACCTGA	47	0
ABCB9	AACTGGCCTTTTTCCCTGGA	53	0
ABCB9	GGAGTTTGCCACAGGTGAGC	37	0
ABCB10	CTTATCGTGCCTGCACCCTC	69	0
ABCB10	GTCCCCTCTGGTCTGAACTT	40	0
ABCB10	AGGGTGCAGGCACGATAAGG	36	0
ABCB10	ATAAGGCGGGTGACAGCCAG	0	0
ABCB10	CCTTATCGTGCCTGCACCCT	105	0
ABCB10	TTCCCAAGTTCAGACCAGAG	72	0
ABCB10	CGTTCCCAAGTTCAGACCAG	211	0
ABCB10	CGTCCCCTCTGGTCTGAACT	77	0
ABCB10	CAAGTTCAGACCAGAGGGGA	84	0
ABCB10	GTTCCCAAGTTCAGACCAGA	0	0
ABCC2	CTACTGATGCTGCCCTTTGT	86	0
ABCC2	ACAAGAGGCCTCTGTAGGAG	0	0
ABCC2	AAAGTACAAGAGGCCTCTGT	0	0
ABCC2	CTGAAAGATGTCAACAGAGC	49	0
ABCC2	GGTGAGTCTCCCTGTCCCTA	73	0
ABCC2	ACCAGTTCCCAAAGTACAAG	96	0
ABCC2	ATGTATGGCCACTCCTACAG	91	0
ABCC2	GACTAAAAAGCCCTAGGGAC	71	0
ABCC2	TCTACTGATGCTGCCCTTTG	383	0
ABCC2	GCCTCTTGTACTTTGGGAAC	184	0
ABCC3	AAGGGCTTCTGTGCTCAATC	79	0
ABCC3	GGGCCGATGAGTCATTGAGT	109	0
ABCC3	TCCCCTACTCAATGACTCAT	58	0

ABCC3	GAGTAGGGGACGTAGGGAGA	88	0
ABCC3	AGTCGAGTCTATTTCCAAGG	17	0
ABCC3	TCAATGACTCATCGGCCCCA	219	0
ABCC3	AGGTTCCAGAGACCACTGGC	44	0
ABCC3	AAGGTCACGTTCCTCTCCGG	266	0
ABCC3	GAGTCGAGTCTATTTCCAAG	143	0
ABCC3	ATCGGCCCCACGGACTTGTT	307	0
ABCC4	AGTCTAGGCGCCGACACAGA	74	0
ABCC4	TCTGTGTCGGCGCCTAGACT	152	0
ABCC4	TCACTCGGGTTCGTGCACGT	36	0
ABCC4	CCGACACAGAAGGTGGAGCA	41	0
ABCC4	TTCACTCGGGTTCGTGCACG	119	0
ABCC4	CTGCTACCCTCTCCATCCAG	0	0
ABCC4	CCCTGCTCCACCTTCTGTGT	231	0
ABCC4	ACACAGAAGGTGGAGCAGGG	54	0
ABCC4	GAATTTCGGAAAGTTTCACT	90	0
ABCC4	TCTCCATCCAGCGGCGCAGG	94	0
ABCC5	AGACTGTCTGGAATCTTCTC	41	0
ABCC5	ACTACATTTCCCAGAAGCGC	62	0
ABCC5	TGTGTGCGAACACAGGGCCA	0	0
ABCC5	CAGATACGATTGCTTGCTCA	179	0
ABCC5	TCGCACACAGTGCTGTTCGT	135	0
ABCC5	AGACAGTCTCGCAGAGGCAC	27	0
ABCC5	CGGTATCTGCGCAGACTGCA	65	0
ABCC5	ATGGAACAGAGCCGGGAGCT	0	0
ABCC5	GCCACAACAGATAAGAACTG	149	0
ABCC5	GATTCCAGACAGTCTCGCAG	392	0
CFTR	CATTGTCCAACACCTGTGGT	128	0
CFTR	TCACTCTTTCTGCCAACCAC	35	0
CFTR	TTTCTGCCAACCACAGGTGT	86	0
CFTR	GTGGTTGGCAGAAAGAGTGA	132	0
CFTR	TATAAATCAAGCCACGTAGT	37	0
CFTR	TTGGAGACTTTGACATGGTT	38	0
CFTR	AGGATAAATTATGGATGTGG	1	0
CFTR	AGGTGTTGGACAATGATTTG	60	0
CFTR	AAATCATTGTCCAACACCTG	0	0

CFTR	CCTACTCAACTGAAAAGTAC	162	0
ABCC8	TCTTTCCAGATCGGGATCTG	41	0
ABCC8	CTCTCCTTCAGCCAAGTACC	263	0
ABCC8	TCTTTGGACACCACAGGTTT	75	0
ABCC8	TTGGCTGAAGGAGAGCTGGG	0	0
ABCC8	AATAGCCTCTATGCTAGCTC	0	0
ABCC8	GTCCCTGTGTAAGCCCTCTC	31	0
ABCC8	CTCTTTGGACACCACAGGTT	69	0
ABCC8	AGGAAACAAGCCCAAACCTG	36	0
ABCC8	GGGCAGTGGTGTGTCTCTAT	29	0
ABCC8	GTACTTGGCTGAAGGAGAGC	35	0
ABCC9	CTCATTTGAGGTACTACTAT	97	0
ABCC9	AAGAAAGCTTCAAGTTGCTT	30	0
ABCC9	CCTCAGCTATCAAAGTAAAA	26	0
ABCC9	TTCTCTGGAAGTAAAAGCTA	162	0
ABCC9	ATGATATAGCCCTCTATTCA	58	0
ABCC9	TTTAGTCAGACACTCATTTG	85	0
ABCC9	TCCTCAGCTATCAAAGTAAA	110	0
ABCC9	CACATGAAAGCATTTTTCTC	125	0
ABCC9	CAGAGAAAAATGCTTTCATG	64	0
ABCC9	CCCTTTTACTTTGATAGCTG	0	0
ABCC10	GCATTATTTGTCCGTGGGGC	0	0
ABCC10	ATTCTAGGGCTGCATGACCT	116	0
ABCC10	TTCTTGAAGAGGTGGGGAGC	46	0
ABCC10	ATTCAAACATACCTGCCCCA	248	0
ABCC10	GCCATCCTCCTGGGATCTAC	114	0
ABCC10	CTCCCCACCTCTTCAAGAAA	57	0
ABCC10	CCCAGTCTCAGATAGATAGA	21	0
ABCC10	GCCTCAACTTTTTTTTGGGG	0	0
ABCC10	CCTCAACTTTTTTTTGGGGG	35	0
ABCC10	CCCCCCCCAAAAAAAAGTTG	0	0
ABCC11	AAGGTCCTCGTTAGCGAAAT	102	0
ABCC11	TAAAGAGCCACGGATCCCAG	124	0
ABCC11	CGAGGTCAGACAGCAGTAGG	202	0
ABCC11	CTAAAGAGCCACGGATCCCA	113	0
ABCC11	TCTAAAGAGCCACGGATCCC	22	0

ABCC11	TCCGTGGCTCTTTAGAGTCA	50	0
ABCC11	CCCTTGACTCTAAAGAGCCA	68	0
ABCC11	CAAAGAAACTCATGGGGCAG	186	0
ABCC11	TGCGTTGATTTTGTCCCTGC	148	0
ABCC11	CCGTGGCTCTTTAGAGTCAA	136	0
ABCC12	CTGACCAGTGGGTATCAGGA	33	0
ABCC12	GGCAGCAGTCACAGAAGTGT	123	0
ABCC12	CCAGTGGGTATCAGGATGGA	29	0
ABCC12	TTTTCTTGGGGCTCTTTCCC	209	0
ABCC12	ACCAGTGGGTATCAGGATGG	214	0
ABCC12	GGAAAGAGCCCCAAGAAAAT	0	0
ABCC12	CTTCAACAACACCACCCCTC	70	0
ABCC12	GTTGAAGTTCTCTGACCAGT	159	0
ABCC12	TGTTGAAGTTCTCTGACCAG	106	0
ABCC12	TCAGATTCTCTCAGATCCCC	41	0
ABCD1	AGCGACAGTGACTCCTCCAC	211	0
ABCD1	GAAGAGTGGGTGGGGATTCT	17	0
ABCD1	GGAAGAGTGGGTGGGGATTC	32	0
ABCD1	CGAGGGGCGAGAACAGGGTG	92	0
ABCD1	CACCTCCCGTTCTCGCCCCT	234	0
ABCD1	GGCCGCGCGGACTAGAGAAG	48	0
ABCD1	GAGAACGGGAGGTGGGGGTG	53	0
ABCD1	CCTCCCGCACCACAGAGACG	136	0
ABCD1	CACGTCTCTGTGGTGCGGGA	68	0
ABCD1	GCGCCTCTTCCTCTCCCGCT	367	0
ABCD2	GGTCGAATCAGGCCATCTGC	39	0
ABCD2	TCAGACTCCGCTGCATCTAC	112	0
ABCD2	TCATTGGCTGTGAGGGCGGT	59	0
ABCD2	ATCATTCCCGGTAGATGCAG	123	0
ABCD2	CGGTTTTGTTCGCCAGCAGA	59	0
ABCD2	ACCGCCCTCACAGCCAATGA	188	0
ABCD2	CAGACTCCGCTGCATCTACC	187	0
ABCD2	GCTTCTGAGAGAATCATTCC	156	0
ABCD2	TCACAGCCAATGAGGGCCT	0	0
ABCD2	GGGAAGCTGCGAGGATTCCA	183	0
ABCD3	ACCCCGGCTTTCACAATAAC	41	0

ABCD3	CTGGAGCCCACTTTGTACTT	0	0
ABCD3	AATAACTGGATGAGCGGGCC	23	0
ABCD3	CTACCTTACTGCACGCAGCG	122	0
ABCD3	ACAAAGTGGGCTCCAGAGCG	149	0
ABCD3	TCCAGTTATTGTGAAAGCCG	0	0
ABCD3	CGCCGGGCCAAAGTACAAAG	0	0
ABCD3	TACCTTACTGCACGCAGCGC	34	0
ABCD3	CTTCAGGGTCTCCGGAAACG	7	0
ABCD3	GCCGGGCCAAAGTACAAAGT	95	0
ABCD4	ACAGCGCCCCCATATTTTCT	138	0
ABCD4	TACAGCGCCCCCATATTTTC	92	0
ABCD4	AAAGTACCAGAGAGTGGACT	77	0
ABCD4	AAAGATCATGCTGGCTTCTC	270	0
ABCD4	GGGAAGTCTCCCAGAAAATA	106	0
ABCD4	AAGATCATGCTGGCTTCTCT	211	0
ABCD4	ACAAAAGCATTAGCCGGGGA	65	0
ABCD4	GATTACAGGAGTAACCCACC	15	0
ABCD4	TATAAATTAGGCTTCCAGCC	37	0
ABCD4	CCGAGCTACTCTGGAGGCTG	0	0
ABCE1	TCAACTGTGTGGTCAGTTCA	382	0
ABCE1	TCAACTGCCGGACTTGGGAC	106	0
ABCE1	AGAATTCAACTGCCGGACTT	41	0
ABCE1	TTAGGGCAGAATTCAACTGC	66	0
ABCE1	TTGTCACTTGCCTGCAAGTT	91	0
ABCE1	CTCGGAAAAGATTGGATTCC	25	0
ABCE1	CTCAACTGTGTGGTCAGTTC	83	0
ABCE1	CTAGATCTCACCTAACTTGC	185	0
ABCE1	ACGGACTGTAAACTTTGGCA	1	0
ABCE1	GAACTGACCACACAGTTGAG	89	0
ABCF2	TCCTTAGGATCCTCCGCGTG	128	0
ABCF2	GGGATACATAGTAGTCCTCA	35	0
ABCF2	GTAAAGCCTGAAGGCTCAGC	0	0
ABCF2	GATAAACCGGCTGAGCCTTC	52	0
ABCF2	CTCTTGGCCGCTGCAACTTG	0	0
ABCF2	GAGCAGCTCTGTTGCGACAT	79	0
ABCF2	GCAAATCCCAGATTGCTCGT	118	0

ABCF2	CTCAAGGCGGGTCTCACTCT	37	0
ABCF2	CAAGAGTGAGACCCGCCTTG	68	0
ABCF2	TGTATCCCACAGACCTCGCG	45	0
ABCF3	GGCTCCAAAATGGGCCTGAC	44	0
ABCF3	CTTCTGAGAGTCAGGGACCG	33	0
ABCF3	CTGAGAAGCCCGTAGGGTCT	0	0
ABCF3	GGGTGAGTCAGCAAAGCTCT	20	0
ABCF3	TTTATCAGCCGCCTGCCTCT	65	0
ABCF3	TTATCAGCCGCCTGCCTCTT	159	0
ABCF3	TGGGAGCTCAAGGTTTATCG	49	0
ABCF3	TGCGGTTCTCGCTGCCTTCT	239	0
ABCF3	TCGAGGGAGCGCATAAAACC	0	0
ABCF3	GCGAAAGCAAAGCACTGAGC	161	0
ABCG1	TCCTTCTGTGGACAGGTACT	23	0
ABCG1	ACTTGGGTCCTTCTGTGGAC	232	0
ABCG1	CTATTACACTGTAGACCTGG	66	0
ABCG1	TTACGCCCAGTGACTTGTGA	1	0
ABCG1	ACCTAGTACCTGTCCACAGA	111	0
ABCG1	GAAGTGAGCAGGGTTACTAA	0	0
ABCG1	TTTACGCCCAGTGACTTGTG	112	0
ABCG1	CTGTTCCCTCACAAGTCACT	63	0
ABCG1	TCTGTTCCCTCACAAGTCAC	163	0
ABCG1	GCAGAAAACAGGAAGTGAGC	0	0
ABCG2	GTATCTTTCTCTTTGTGCCG	41	0
ABCG2	GTCAGCGTGGGATCCTCTTC	0	0
ABCG2	ATGCTGTCATCAGAATTCCG	0	0
ABCG2	CTAATGGTTCCAGTCAGCGT	41	0
ABCG2	TCTTCTGGCATTCCTAGCAA	64	0
ABCG2	TAGCAAGGGGCTAGAAGAAG	0	0
ABCG2	CTAGCAAGGGGCTAGAAGAA	133	0
ABCG2	GAAGAGGATCCCACGCTGAC	91	0
ABCG2	CCACGCTGACTGGAACCATT	74	0
ABCG2	CCTAATGGTTCCAGTCAGCG	38	0
ABCG4	CCTCGTTTGGGGATTCCTGG	0	0
ABCG4	CTTCCAGGGACTTGTCCCCA	0	0
ABCG4	TTCAAAAACGCAGGAGCCTT	38	0

ABCG4	CTGGAACTGAGGGAAATTGT	76	0
ABCG4	GAAAGCCTTTGCACCTCGTT	85	0
ABCG4	CCTCCAGGAATCCCCAAACG	60	0
ABCG4	AAGCCTTTGCACCTCGTTTG	133	0
ABCG4	AAGTCCCTGGAAGACAGATT	42	0
ABCG4	GGAGGCCTGCGCTCATTCAT	171	0
ABCG4	AATCCCCAAACGAGGTGCAA	97	0
ABCG5	TCTAAGAGAGCTGCAGCCCA	271	0
ABCG5	GTGAGTGAGCAATGGGAAGT	132	0
ABCG5	AGAACACACACGTTTGTAGG	21	0
ABCG5	CGCGTCCTTATCTTGACAGT	564	0
ABCG5	TGTAGGTGGGCTTGCCAGCA	234	0
ABCG5	GATAAGGACGCGCTGGCTAA	375	0
ABCG5	TACCTCGGTGAGTGAGCAAT	136	0
ABCG5	CTCACTCACCGAGGTATCCT	147	0
ABCG5	GCGCGTCCTTATCTTGACAG	33	0
ABCG5	CTGTCAAGATAAGGACGCGC	74	0
ABCG8	CGCGTCCTTATCTTGACAGT	564	0
ABCG8	AGAACACACACGTTTGTAGG	21	0
ABCG8	TCTCATCTTTGACCCCCGGA	29	0
ABCG8	TGGGCAAATTTTCTGGTGGC	30	0
ABCG8	GATAAGGACGCGCTGGCTAA	375	0
ABCG8	GACCTCTCATCTTTGACCCC	109	0
ABCG8	CTCTCATCTTTGACCCCCGG	86	0
ABCG8	TTCTGCCCACTGTCAAGATA	318	0
ABCG8	CAACTGAAGCCACTCTGGGG	0	0
ABCG8	TCCCCAACTGAAGCCACTCT	199	0
ATP1A1	CAGATCGATTCCAGCTATGA	35	0
ATP1A1	AGGAGGGCGCTTGAAACTTC	0	0
ATP1A1	TCAGAGTGCCTCCTGTGACT	27	0
ATP1A1	GGAGCGGGAAGTGGATAAAG	291	0
ATP1A1	TAGCTGGAATCGATCTGAAC	204	0
ATP1A1	TCTCGAAGAAAGAGAGGTGG	0	0
ATP1A1	AGCTATGACGGAGCGGGAAG	52	0
ATP1A1	CGATTCCAGCTATGACGGAG	1	0
ATP1A1	AATCCCCAAACTGGACAAGC	102	0

ATP1A1	GAATCTCGAAGAAAGAGAGG	0	0
ATP1A2	GTGGCACTCACAACTGAGAG	0	0
ATP1A2	TAGACAGCCCGTTTGTCCCA	82	0
ATP1A2	CCCCTTCAGCCAGATCCAAG	74	0
ATP1A2	TCCCCTTCAGCCAGATCCAA	124	0
ATP1A2	GGGATATGCCCCCTTGGATC	36	0
ATP1A2	ACAAACGTTTCTTTCGGAGG	32	0
ATP1A2	AAACGTTTCTTTCGGAGGAG	93	0
ATP1A2	GTGGGACAAACGGGCTGTCT	21	0
ATP1A2	CCCCTTGGATCTGGCTGAAG	34	0
ATP1A2	CCCCCTTGGATCTGGCTGAA	64	0
ATP1A3	TTCCCAGAGACCTCCGCAAT	177	0
ATP1A3	CCCAGAGACCTCCGCAATAG	73	0
ATP1A3	ATCACCGAGAAGGGTACGCT	85	0
ATP1A3	TCCCAGAGACCTCCGCAATA	106	0
ATP1A3	TACTGCAGCTGTCCTCCCCT	366	0
ATP1A3	TGGAGTAGGGCCTCCAAGAA	166	0
ATP1A3	TCCCCTATTGCGGAGGTCTC	27	0
ATP1A3	GGAGGACAGCTGCAGTACCA	159	0
ATP1A3	GGCCCTACTCCATATTGAGG	142	0
ATP1A3	TCACCGAGAAGGGTACGCTG	0	0
ATP1A4	CATCGCCAAGGCAAGACTTC	227	0
ATP1A4	GGCCTTTGTGATGCCAGACC	38	0
ATP1A4	GTCCAGGTCTGCTGTCGCAA	83	0
ATP1A4	TGCAGACTTTGGAGGCTTAG	45	0
ATP1A4	CTGTCGCAAAGGCCTCATGA	40	0
ATP1A4	AGTCTCAGTCTTTGGGTTTG	160	0
ATP1A4	AAATAAAGCGGGTATTCGGC	26	0
ATP1A4	AATAAAGCGGGTATTCGGCC	36	0
ATP1A4	GACTGAGACTTCTAGGTTCT	51	0
ATP1A4	AGAATCCAGAAGTCTTGCCT	103	0
ATP1B1	GAAAACAACCAGACTTTGCC	76	0
ATP1B1	TTGTGGATCAGTCAGTAGGT	0	0
ATP1B1	TGTGGATCAGTCAGTAGGTT	57	0
ATP1B1	ATTTTGTGCAGCCTGTTGCT	62	0
ATP1B1	TGTGCAGCCTGTTGCTTGGT	69	0

ATP1B1	ACAGTTGTGGATCAGTCAGT	29	0
ATP1B1	GCCCGAAACCTGGCAAAGTC	63	0
ATP1B1	CTATGAGTCGTGACTGCGTG	32	0
ATP1B1	GTGACTGCGTGAGGGAAGGA	40	0
ATP1B1	TATGAGTCGTGACTGCGTGA	99	0
ATP1B2	TGGACGGCAGTTTGGATGTG	150	0
ATP1B2	GATGTGAGGTGTTGAGAAAC	35	0
ATP1B2	AGTAACATCCAGTGGAGCAC	18	0
ATP1B2	TGAGGATGTGGACGGCAGTT	51	0
ATP1B2	GGTGTTGAGAAACTGGGCTA	0	0
ATP1B2	GGCCCATGCTCATAGTTGTT	68	0
ATP1B2	AACCCACTCCCTCCAGTCCA	162	0
ATP1B2	CAGCTCCATGAATGTTTGGG	25	0
ATP1B2	CAAATCTTGCCGGTTTTCCT	74	0
ATP1B2	TCAGCTCCATGAATGTTTGG	72	0
ATP1B3	CCTCGCTTGGCTCCCTTGAA	113	0
ATP1B3	CACAGGAAAGGAACACTCGT	72	0
ATP1B3	GGGTTAAGAGTAGGAGCGTC	1	0
ATP1B3	AACGAGTGTTCCTTTCCTGT	97	0
ATP1B3	GCAGTGCACCGTGTACATTG	68	0
ATP1B3	GGAGCGTCTGGGCAAAAGCT	0	0
ATP1B3	ACGTGCTTTCCCAAGGGTCC	70	0
ATP1B3	AAAAGCCGGCGACACTACAC	72	0
ATP1B3	AGGAAAGGAACACTCGTTGG	175	0
ATP1B3	CGGGGCTCCCACAATGTACA	165	0
FXYD2	GGTTGGTTAATTCCAGGGCC	52	0
FXYD2	AGGGGGGTACATATGGAAAA	68	0
FXYD2	ATATGTACCCCCCTGGGCCA	100	0
FXYD2	CCCCTCCTGGTACTAAGCTC	38	0
FXYD2	GCCCCAAGAGTGGAAAGAGG	0	0
FXYD2	GCACATTAGGAGTCCGCACT	74	0
FXYD2	TACCAAGATGGGCCTCTGTG	66	0
FXYD2	AGGCCCCAGGCTACCAAGAT	192	0
FXYD2	CCCGAGCTTAGTACCAGGAG	0	0
FXYD2	CACTCTTGGGGCCACCTATT	275	0
ATP1B4	AGTTGGATATCAGTGCATGC	294	0

ATP1B4	TTGGATATCAGTGCATGCAG	130	0
ATP1B4	TTGGCTCCTTCCCTGAATCA	84	0
ATP1B4	CTCCAGCGCTTGAGATATGA	35	0
ATP1B4	GAATCAAGGGAAAGCCCTTA	20	0
ATP1B4	AGCTCATCATTACAAGGAGG	44	0
ATP1B4	ACAGGAGACACTGGTTGCTG	102	0
ATP1B4	ACAAGGAGGGGGAGATATTC	37	0
ATP1B4	AGACATATAAAGGGGGCGGG	0	0
ATP1B4	GCAAGCTCATCATTACAAGG	52	0
ATP2A1	AGAGGGCTCCGGGAAGAACT	0	0
ATP2A1	TGGGAAATTCAATGCAGCTG	125	0
ATP2A1	GATATTTCTGCTGACAGAGG	0	0
ATP2A1	CAGAGATACCCCAAGCCTTT	35	0
ATP2A1	ATCAGTCCCCCAAAGGCTTG	155	0
ATP2A1	CTTCAGCAAATGGGGTGAGG	0	0
ATP2A1	CACCCCATTTGCTGAAGCAG	200	0
ATP2A1	GAGACTGCGGCAATGGAAAC	204	0
ATP2A1	GTCACTCATGAGGGACGCAA	70	0
ATP2A1	TGCTGACAGAGGGGGAACAG	53	0
ATP2A2	TAGCTGACGCAGCGCTTGAT	79	0
ATP2A2	GTTCCCGAGGCGACAGATGA	31	0
ATP2A2	GCCACACAGCAGCTAGACTT	125	0
ATP2A2	GCAGCTAGACTTGGGCTAGG	310	0
ATP2A2	GCCCAAGTCTAGCTGCTGTG	132	0
ATP2A2	TTCCCTCGACAGGAGAAAAG	36	0
ATP2A2	GAGGCGACAGATGAAGGATT	0	0
ATP2A2	TCGCTGTGGCTTTCAGATGA	212	0
ATP2A2	GCGCTTGATAGGTGTCTGCA	113	0
ATP2A2	GTCCCCTTTTCTCCTGTCGA	150	0
ATP2A3	CACTTCGCAGAATTCGAGCT	125	0
ATP2A3	TCACTTCGCAGAATTCGAGC	0	0
ATP2A3	ATTCTGCGAAGTGACTCCAG	45	0
ATP2A3	TTCTGCGAAGTGACTCCAGA	117	0
ATP2A3	CAGAATTCGAGCTGGGAAGC	56	0
ATP2A3	TTCCCCTCTGCAGAATGGGC	141	0
ATP2A3	TTTCCCCTCTGCAGAATGGG	46	0

ATP2A3	CAGTTTCCCCTCTGCAGAAT	211	0
ATP2A3	TCAGTTTCCCCTCTGCAGAA	36	0
ATP2A3	TCCGCGGAGCTGCTAGGGGT	131	0
ATP2B1	GAAGAAGGAGTAACACCTCA	117	0
ATP2B1	AAGAAGGAGTAACACCTCAT	107	0
ATP2B1	CTCTTATCACTAGAAGAAGA	25	0
ATP2B1	GCACTTTAAAAGCACAAGAC	37	0
ATP2B1	AAAACAACTAGCATAGTGCT	0	0
ATP2B1	CATTACAATTTAATCCCATG	47	0
ATP2B1	CATATTTTCTAAATGTCACC	0	0
ATP2B1	AATTATCTACTTTAGCTACC	0	0
ATP2B1	ACAATAACAGTAATACTGAG	40	0
ATP2B2	TCCTGAGCACCTGTCCCATG	110	0
ATP2B2	AAGCACAGAGTGAGTGACAA	40	0
ATP2B2	AGGTTGCTGTGCGTGTGCCT	32	0
ATP2B2	TAGGTTGCTGTGCGTGTGCC	0	0
ATP2B2	GTGCCACAGCTGACCAGTGA	134	0
ATP2B2	TGACCAGTGAGGGTGTGCTG	33	0
ATP2B2	CAGTGGTGGTGAGCTCCAAG	183	0
ATP2B2	CAAGAGGTGTCCTCCGGTGT	176	0
ATP2B2	GGCATCTACATGGTCAGGGG	0	0
ATP2B2	GAACTGGCATCTACATGGTC	107	0
ATP2B3	CAGCCTCAGTCGCAAGCTCA	252	0
ATP2B3	GCGTCTCCCCAACAGTAAAA	46	0
ATP2B3	GTTGGGGAGACGCACCTAGA	153	0
ATP2B3	TGTTGGGGAGACGCACCTAG	0	0
ATP2B3	TCTGTGCCCCATTTTACTGT	79	0
ATP2B3	CTGTGCCCCATTTTACTGTT	257	0
ATP2B3	GGATGGAAACTGGGGGACCA	45	0
ATP2B3	GTAAAATGGGGCACAGAGCC	83	0
ATP2B3	GTGGGTGGGGATGGAAACTG	17	0
ATP2B3	GGTGCTCTCTCTGCACTCAC	156	0
ATP2B4	CCTGGATCTAAGTGAGTAAG	96	0
ATP2B4	GGCGGTATTCAACGGTTGCT	81	0
ATP2B4	TGTCTGAATAGGGGGTTCCT	34	0
ATP2B4	CTCCCCTACACCTTATATAC	122	0

ATP2B4	ATATAAGGTGTAGGGGAGGA	0	0
ATP2B4	CCCCCTATTCAGACAGGAGC	106	0
ATP2B4	GTCCTGGATCTAAGTGAGTA	104	0
ATP2B4	ACTGTCAGTATACCTCTGGT	87	0
ATP2B4	CCCCTTACTCACTTAGATCC	69	0
ATP2B4	ATTCAACGGTTGCTGGGCGG	100	0
ATP2C1	CATACCCACAGACTCTCGTG	40	0
ATP2C1	TTAAGCCTCACGAGAGTCTG	139	0
ATP2C1	TAAGCCTCACGAGAGTCTGT	48	0
ATP2C1	CCTGAATTCCTATTGCCCTT	75	0
ATP2C1	AGCTCAGTTGCGGTCTGAGC	47	0
ATP2C1	CCAAAGGGCAATAGGAATTC	43	0
ATP2C1	CTCACGAGAGTCTGTGGGTA	39	0
ATP2C1	ACAGACACACAGCTCAGTTG	34	0
ATP2C1	CTTAAACAGGGCATGAGATG	36	0
ATP2C1	GGCAATAGGAATTCAGGGGC	15	0
ATP4A	GGACCAGTCCTGATATACCC	0	0
ATP4A	TCTCAGGGCAGGTTGACATG	152	0
ATP4A	TTCTCCTATGGCTCCATCAC	238	0
ATP4A	AGGTTGACATGGGGGGATCT	72	0
ATP4A	TGTTGCTGCAGCGGTTCCCA	176	0
ATP4A	ATCTCAGGGCAGGTTGACAT	106	0
ATP4A	TTGTTGCTGCAGCGGTTCCC	121	0
ATP4A	ATATACCCAGGGAGGGCGGT	0	0
ATP4A	GGGGTTGAGTCATTCTCCTA	151	0
ATP4A	TTGTCCCCTCAAAGGGTTAT	91	0
ATP4B	GAGGAAACTATAAAGCCCAG	82	0
ATP4B	ATTGCGGTGAGGGCACGTTC	0	0
ATP4B	CAATCTCCCTCCTGAAGGAG	71	0
ATP4B	AGCTTTGTCTGCTCTGATTG	117	0
ATP4B	GAAACTATAAAGCCCAGAGG	0	0
ATP4B	TGGGCATCTGGCCTCAGTCT	0	0
ATP4B	GTCTGCTCTGATTGCGGTGA	58	0
ATP4B	ACCCACTGTGTCCACGACCT	202	0
ATP4B	GACCTCAATCTCCCTCCTGA	23	0
ATP4B	CCAGAGGTCAAAGGCATCGC	105	0

ATP5A1	CGCCGCCACTCTGCATTTTT	132	0
ATP5A1	TTGCTACTCGTGCTAAACCA	0	0
ATP5A1	ACCGACACTATTAATCGCCC	343	0
ATP5A1	ACAGTGAACAGCTAAACCCC	27	0
ATP5A1	CCTCATCCTCTAGCACCAGA	81	0
ATP5A1	TCTAGCACCAGATGGGGCCT	119	0
ATP5A1	CAAAAAATGCAGAGTGGCGG	0	0
ATP5A1	TCATCCTCTAGCACCAGATG	150	0
ATP5A1	AGTCGACCTTGTGGTTGCCC	107	0
ATP5A1	GAACAGCTAAACCCCGGGAC	0	46
ATP5B	AGTCCTTTGTGTGACGGAAA	42	0
ATP5B	TGGGGTCTTGTGCATGCGGA	0	0
ATP5B	GTTCAGTACCCCTATTCAAC	112	0
ATP5B	CAGGCCTCTTTCCGCTTGTA	179	0
ATP5B	TCAGGCCAGTTAAAGGTCAT	74	0
ATP5B	AGGACTTAACACACAAGGAG	25	0
ATP5B	GTTGACCTTCCGGTTGAATA	59	0
ATP5B	CGTTCCGTACAAGCGGAAAG	0	0
ATP5B	AGTACCCCTATTCAACCGGA	126	0
ATP5B	GTACGGAACGCGTGTCCAAG	35	0
ATP5C1	GTTTCTTCGCGCTACGGCGA	81	0
ATP5C1	GGCACTGCTTCTGGCACATC	199	0
ATP5C1	TTTCTTCGCGCTACGGCGAG	34	0
ATP5C1	CCACGGCAGCGATCACTTTC	257	0
ATP5C1	CCAGAAAGTGATCGCTGCCG	37	0
ATP5C1	AGATGTGCCAGAAGCAGTGC	121	0
ATP5C1	AGTACTCAGCCAGCCGGAAA	40	0
ATP5C1	AATCCCAGCTGAGAGGCGGA	64	0
ATP5C1	CGCCAACAGGATCAAGTCCA	0	0
ATP5C1	TTCCGGCTGGCTGAGTACTG	144	0
ATP5D	TGGGGCGCTCATTGGATGCA	173	0
ATP5D	AGGGGGTGGTCAGATAAGGG	0	0
ATP5D	GTTGCAGTTTTGTTCCGGCC	115	0
ATP5D	CCCCCAGAATACCTCACGAC	56	0
ATP5D	TGCCCCGTCGTGAGGTATTC	209	0
ATP5D	CCCGTCGTGAGGTATTCTGG	127	0

ATP5D	ACCCCCAGAATACCTCACGA	147	0
ATP5D	GCCCCGTCGTGAGGTATTCT	39	0
ATP5D	AAGGGCGGTGAAACCGTCCT	26	0
ATP5D	TTGCAGTTTTGTTCCGGCCC	319	0
ATP5E	CGGGAGCTCTTGGAGCCAAT	116	0
ATP5E	GCCGGGGTAATACGGAAGGT	34	0
ATP5E	TATTGGCTCCAAGAGCTCCC	26	0
ATP5E	AGCTCTTGGAGCCAATAGGT	32	0
ATP5E	CTCAGTGGTCCTTAGCGAGC	87	0
ATP5E	TCAGTGGTCCTTAGCGAGCG	87	0
ATP5E	GGGGTAATACGGAAGGTCGG	66	0
ATP5E	ACCTTCCGTATTACCCCGGC	109	0
ATP5E	TTGCGCCCGCTTCAGGGAAA	27	0
ATP5E	CGGGGTAATACGGAAGGTCG	17	0
ATP5F1	ATTCTAGTGGCCTCCGATTC	84	0
ATP5F1	ACAAGCTGTCAGCGCGTGAA	200	0
ATP5F1	CTCCATTCTACCGTTCCACC	164	0
ATP5F1	TAGTGACTCACCTGAATCGG	78	0
ATP5F1	TCCATTCTACCGTTCCACCC	107	0
ATP5F1	GGATAGTGACTCACCTGAAT	240	0
ATP5F1	CCATTCTACCGTTCCACCCG	103	0
ATP5F1	TGGCTGACCTCACTTGGGTT	0	0
ATP5F1	AGCTTGTACCCCCCGTAGAT	80	0
ATP5F1	GGTAGAAGGGTTCGCCTCCA	218	0
ATP5G1	CATCGATTCCCCACTCTTAT	189	0
ATP5G1	ATTGGTAGTTGCGCTGAGGG	39	0
ATP5G1	TTTGGCCCCGAGCCTTCATT	494	0
ATP5G1	TGCGGAAAGCCAATAAGAGT	88	0
ATP5G1	CCATCTTGTGCGCGAGGTTA	78	0
ATP5G1	TGGCAATTCCCGCCTCCATG	0	0
ATP5G1	GAATCGATGACGTCAACCAA	148	0
ATP5G1	GAAACGCTCGACCATAGAGT	29	0
ATP5G1	TAATATCCCCGCGTCCCCAT	112	0
ATP5G1	GAGCCTTCATTCGGGCGCTT	126	0
ATP5G2	CCTAGGAAGATGATCTCCTT	207	0
ATP5G2	AGTCTATTCTTGCTGCCCCA	217	0

ATP5G2	GGTCAAGAAGTGTAAGACTG	0	0
ATP5G2	TCCTAGGAAGATGATCTCCT	41	0
ATP5G2	TCTGTTTGGGGGAGAAACAA	29	0
ATP5G2	CCCAAGGAGATCATCTTCCT	98	0
ATP5G2	GGGGCAGCAAGAATAGACTA	116	0
ATP5G2	CTGTTTGGGGGAGAAACAAT	43	0
ATP5G2	TGGGGCAGCAAGAATAGACT	142	0
ATP5G2	CTAGGAAGATGATCTCCTTG	73	0
ATP5G3	CTTCTTTGAGATCTACGTGG	152	0
ATP5G3	TTTTAGCACCAGAGTCCATG	70	0
ATP5G3	CTACTTCTTTGAGATCTACG	41	0
ATP5G3	TTAAAAATTAGCAGGTGTGG	21	0
ATP5G3	ATAAAATGCCTCATGGACTC	84	0
ATP5G3	AATTTAAAAATTAGCAGGTG	32	0
ATP5G3	TTTACAAATAAAATGCCTCA	63	0
ATP5G3	CATCTCTTTTTTTTGAGACA	17	0
ATP5G3	TAGTAATACATAACTAAAAC	0	0
ATP5G3	ATTTGAAGCTATTAAGTTTT	25	0
ATP5H	AAAAAATTAGCCAGGCATGG	0	0
ATP5H	GGCTTGAGCTTGGGAGGCAG	151	0
ATP5H	AGCCAGGCATGGTGGTGCTG	50	0
ATP5H	TTTTGTCATATTGCTCAGGC	179	0
ATP5H	CGAAAATTTATCAGGTGTGG	0	0
ATP5H	GTGGGAGGAAGGCTTGAGCC	46	0
ATP5H	CCCAGTTACTCGGGGGGATG	0	0
ATP5H	AAATTTATCAGGTGTGGTGG	65	0
ATP5H	AAAAAAGAAGAGCAGAGAGG	22	0
ATP5H	TGGGAGGAAGGCTTGAGCCC	43	0
ATP5I	TGCAGGTTGTGCTTCCGGTG	36	0
ATP5I	TCCGCTCATCAAGGTGATCC	113	0
ATP5I	CCGTCTGCAGGTTGTGCTTC	83	0
ATP5I	GCAGGTCTCTCCGCTCATCA	372	0
ATP5I	TGCGGAGGTCAGGGACAAGA	198	0
ATP5I	ATGAGCGGAGAGACCTGCAC	38	0
ATP5I	CGCACAAGCCAGCAAAGCCT	68	0
ATP5I	GCCTGGATCACCTTGATGAG	23	0

ATP5I	CCGGAAGCACAACCTGCAGA	121	0
ATP5I	CAAGGCTTTGCTGGCTTGTG	118	0
ATP5J	CTTCGATAACCCTAACGAAC	0	0
ATP5J	GGGTCCAGGACTACAACTCC	110	0
ATP5J	CAGGACTACAACTCCCGGCA	43	0
ATP5J	TCGGAGGGGAGCTTGAACTA	33	0
ATP5J	AGTTGTAGTCCTGGACCCGA	0	0
ATP5J	CGGAGGGGAGCTTGAACTAG	66	0
ATP5J	GTACGCATGCGCTCTTTGAG	151	0
ATP5J	AGTCAGCGTCCTGTTCGTTA	67	0
ATP5J	GAGTCAGCGTCCTGTTCGTT	49	0
ATP5J	GAACAGGACGCTGACTCGGA	37	0
ATP5J2	ATGGCCTTCTAGTAGTTCCA	198	0
ATP5J2	CATGGCCTTCTAGTAGTTCC	129	0
ATP5J2	TCTGCTCTACGCCCCTGGAT	99	0
ATP5J2	CCTGGGCTCTCTACCTTAAA	76	0
ATP5J2	CCTGGACAGCAAAAATCCGA	31	0
ATP5J2	GGTCACAACTGCGAACACCC	97	0
ATP5J2	GGCTCTCTACCTTAAAAGGT	101	0
ATP5J2	CGGCCTCTTCCCACCTTTTA	224	0
ATP5J2	ACTAGAAGGCCATGAAGCGA	35	0
ATP5J2	GGGCTCTCTACCTTAAAAGG	0	0
ATP5L	TACAGCACTTTGGGGAGATT	73	0
ATP5L	CCAAAGTGCTGTATCACGAT	75	0
ATP5L	CCAATCGTGATACAGCACTT	122	0
ATP5L	AAAGGGCTATGGGTTGAGCC	3	0
ATP5L	ATACAGCACTTTGGGGAGAT	51	0
ATP5L	TTTAAAAGGGCTATGGGGCC	15	0
ATP5L	ACAACAATCGCTGGAACCCG	42	0
ATP5L	CAACAATCGCTGGAACCCGG	135	0
ATP5L	ACAGTCTCGCTCTGTCGCCC	0	0
ATP5L	CAATCGTGATACAGCACTTT	261	0
ATP5O	AACTACAACTCCCAGCCCGA	50	0
ATP5O	GAACTACAACTCCCAGCCCG	0	0
ATP5O	ACACTTCCCAGAGAACCTAG	226	0
ATP5O	GCTGTAGGTCAAACCCGAGT	0	0

ATP5O	ATTCTACAGTGAGCGCCGCT	66	0
ATP5O	GATGCAGGCTTTAGCATGCT	108	0
ATP5O	GCGTAACCGCTAGGTTCTCT	44	0
ATP5O	GGCGTAACCGCTAGGTTCTC	0	0
ATP5O	AGTGGTAAGGGCTGGACCCT	100	0
ATP5O	CAGGCTTTAGCATGCTGGGA	30	0
ATP6AP1	GGTTGGCTGTACGTGTCTCC	106	0
ATP6AP1	GCACTTGGTCCAATTACCTG	69	0
ATP6AP1	CCGACTAGGACGAGACAAGA	0	0
ATP6AP1	GACCAAGTGCATGCCACTCG	325	0
ATP6AP1	CCCTCTTGTCTCGTCCTAGT	39	0
ATP6AP1	ACCGACTAGGACGAGACAAG	76	0
ATP6AP1	CTCGTCCTAGTCGGTCTAGC	46	0
ATP6AP1	CTCACGCTCCGAGTGGGAAA	111	0
ATP6AP1	CGTACAGCCAACCAGTGAGA	58	0
ATP6AP1	TCGTCCTAGTCGGTCTAGCT	44	0
ATP6V0A1	AATGAGGTCATTGCGGGGGC	45	0
ATP6V0A1	ATACAGCACAGGGGCCAACA	157	0
ATP6V0A1	CTTACCCCTTATACTGGGGT	28	0
ATP6V0A1	CAACTGCAATGAGGTCATTG	249	0
ATP6V0A1	TGTGCTGTATCTCCACGCTG	78	0
ATP6V0A1	CAGCGTGGAGATACAGCACA	129	0
ATP6V0A1	AACTGCAATGAGGTCATTGC	54	0
ATP6V0A1	AGCGTGGAGATACAGCACAG	107	0
ATP6V0A1	ACAACCTACCCCAGTATAAG	76	0
ATP6V0A1	ACCTCATTGCAGTTGGCCAA	32	0
ATP6V0A2	CTAGGAACGGGTGAGCTTCG	76	0
ATP6V0A2	AACCTCGGGCGTCCATTGGT	194	0
ATP6V0A2	CGAGACTCCTCCAGGAAAGC	0	0
ATP6V0A2	CAGGGTCGTGCACTAGGAAC	74	0
ATP6V0A2	ACGCAGAGCTAAATCCCGCG	181	0
ATP6V0A2	ATTTAGCTCTGCGTCCAGTC	129	1560
ATP6V0A2	ACAGGGTCGTGCACTAGGAA	195	0
ATP6V0A2	GGAATCTCACGCAGTCCCAC	88	0
ATP6V0A2	AGACTCCTCCAGGAAAGCCG	0	0
ATP6V0A2	GAGACTCCTCCAGGAAAGCC	0	0

TCIRG1	CATTCGCAGGCTCATGGGCT	0	0
TCIRG1	TTAATCCCGGAGAGGAGGCC	0	0
TCIRG1	ATTCGCAGGCTCATGGGCTT	134	0
TCIRG1	TCTCATCTCCTATCCTGAGC	121	0
TCIRG1	TATCCTGAGCTGGGCCTGAG	106	0
TCIRG1	TGAGGCGCTTAATCCCGGAG	80	0
TCIRG1	TCTCCGGGATTAAGCGCCTC	100	0
TCIRG1	CCATGAGCCTGCGAATGTTG	33	0
TCIRG1	GGTCCTGAGGCGCTTAATCC	80	0
TCIRG1	CTTGTGCGCCTCTGGCTGAT	67	0
ATP6V0A4	GCTGGAGTCTCACCCATCAG	169	0
ATP6V0A4	TTGGCCTTTGCCACAAAGAA	84	0
ATP6V0A4	TGCAAAGCACGACAGCCCAG	108	0
ATP6V0A4	GGTTGGTCCTGCGCAAAGGA	0	0
ATP6V0A4	GAGTATCTGATCTCCGTCTC	0	0
ATP6V0A4	TGCTGGAGTCTCACCCATCA	163	0
ATP6V0A4	GAAGATTCTTTGGCCAGGGT	134	0
ATP6V0A4	TCATCTGAAGGATTAGGCAG	135	0
ATP6V0A4	TATTCATGGCCAGGCTGGGA	119	0
ATP6V0A4	GGGGGAAGATTCTTTGGCCA	100	0
ATP6V0B	CAGTGAGGTCGCGGTATGGT	81	0
ATP6V0B	TGATGGCTCCCATTTCATTC	150	0
ATP6V0B	AAGCATCACCAGGACATCGC	201	0
ATP6V0B	AGAGGAGCGTTTAAGTGGGG	0	0
ATP6V0B	GATGAGCCGCATGTATAGTT	214	0
ATP6V0B	AGCCATCAGGGCATTTCGAC	49	0
ATP6V0B	TAAGCATCACCAGGACATCG	82	0
ATP6V0B	AGCAGAGGAGCGTTTAAGTG	95	0
ATP6V0B	GCCATCAGGGCATTTCGACA	44	0
ATP6V0B	ACCCTGTCGAAATGCCCTGA	273	0
ATP6V0C	GTACAAAGAAAAGCGTCGCC	57	0
ATP6V0C	TACCAATCCTGGGCGAGCTA	174	0
ATP6V0C	GGTACAAAGAAAAGCGTCGC	114	0
ATP6V0C	CTTCATTAGCATACGGGGCG	27	0
ATP6V0C	GTCTGCAGGTGTTGGGTTCC	62	0
ATP6V0C	TAGCTCGCCCAGGATTGGTA	149	0

ATP6V0C	GCTTCATTAGCATACGGGGC	91	0
ATP6V0C	GTGTGCTTCATTAGCATACG	123	0
ATP6V0C	TGCTTCATTAGCATACGGGG	0	0
ATP6V0C	ACCAATCCTGGGCGAGCTAA	181	0
ATP6V0D1	GCTCTGTTGGAAACCTCTAC	0	0
ATP6V0D1	CTGAGAAGACTCTGCTCTGT	75	0
ATP6V0D1	CTCTCCACATTGTGGCTAGA	136	0
ATP6V0D1	GCATGGGATCAAGTTACCCA	223	0
ATP6V0D1	TAGTTCCTAGCGCAGTCTGA	86	0
ATP6V0D1	TTGAACATAGTCCCGTGGGC	127	0
ATP6V0D1	AGGACACATTCCAGATGGAC	132	0
ATP6V0D1	GAAGTTTGAACATAGTCCCG	35	0
ATP6V0D1	AGCCCCTTCTAGCCACAATG	156	0
ATP6V0D1	CTCCACATTGTGGCTAGAAG	36	0
ATP6V0D2	ATTCAGCTGACTGCTTAGCT	101	0
ATP6V0D2	AAGCTAAGCAGTCAGCTGAA	145	0
ATP6V0D2	CCGTTTAAGGCAGCTCTCTA	70	0
ATP6V0D2	CATAGCTTTACATCCCTGAG	96	0
ATP6V0D2	AGGCAACCTTGCTGCAGCTC	53	0
ATP6V0D2	AGGGGCACTATCTCCGTTTA	104	0
ATP6V0D2	TTCTATGACCCTTGTCACCA	73	0
ATP6V0D2	CCTTAGAGAGCTGCCTTAAA	197	0
ATP6V0D2	GTAGAAGGAGGGGATAGAGC	22	0
ATP6V0D2	AGGAGGGGATAGAGCAGGAG	114	0
ATP6V1A	TCTTAAAACTTGAGGCGTGC	96	0
ATP6V1A	GGAGCTCGACGTCATGTGAC	0	0
ATP6V1A	TCGACGTCATGTGACTGGTT	218	0
ATP6V1A	AGAGCCTGGAATTTTACCGG	136	0
ATP6V1A	ATTTTACCGGCGGACTCCAG	91	0
ATP6V1A	TGGAGGGTAGCCAATCTATC	111	0
ATP6V1A	AAAACTTGAGGCGTGCTGGA	165	0
ATP6V1A	TTGGCCCAAACCTCAGAGCC	189	0
ATP6V1A	TGACGTTCACGCCACAATCG	35	0
ATP6V1A	TCCCAGCGCTCTACATCTCG	200	0
ATP6V1B1	GTTTACTGTCAACCTAGAGG	0	0
ATP6V1B1	TCCTCCAGCTCTCAGGTGAC	0	0

ATP6V1B1	CATACAGTTTGGGGTTGGCG	80	0
ATP6V1B1	TACTGTCAACCTAGAGGAGG	96	0
ATP6V1B1	GACCTGCACAGCTTGGTGTG	170	0
ATP6V1B1	GAACCGCCTAGCACAGAAGA	62	0
ATP6V1B1	TCATACAGTTTGGGGTTGGC	45	0
ATP6V1B1	CACACCAAGCTGTGCAGGTC	45	0
ATP6V1B1	TGACAGCACCTGATGGTCCA	231	0
ATP6V1B1	AGCCTGCCAGAAGTTGGGTG	27	0
ATP6V1B2	CAGACCTGCTTGCGAGCAAG	23	0
ATP6V1B2	TCACGAAAGCCCATTCGAAA	33	0
ATP6V1B2	AGATATACCAAGGCGGCCCG	47	0
ATP6V1B2	CCGCTTGTGCGCCTCTCTTT	124	0
ATP6V1B2	TTGTGCGCCTCTCTTTCGGC	245	0
ATP6V1B2	CACGCGCAGATATACCAAGG	0	0
ATP6V1B2	AGCTCCTCTTGCTCGCAAGC	123	0
ATP6V1B2	CTCTTGCTCGCAAGCAGGTC	131	0
ATP6V1B2	CCGAAAGAGAGGCGCACAAG	0	0
ATP6V1B2	TGGGCTTTCGTGACGCTGAA	215	0
ATP6V1C1	GGCAAGGGGACCCTATATTC	88	0
ATP6V1C1	CAGCGATCAGCCCCGAATAT	39	0
ATP6V1C1	AGCGATCAGCCCCGAATATA	38	0
ATP6V1C1	TGATCGCTGTTGTTCCAGAG	285	0
ATP6V1C1	GTAAACGTCAGCGCCACTGC	0	0
ATP6V1C1	TGACCCGCAGAAACCTGCAG	34	0
ATP6V1C1	ATTGTCTCCAACCCCCTACA	165	1
ATP6V1C1	GCGCTGACGTTTACAAGTGG	0	0
ATP6V1C1	GCTTCTTCCAGTGTTGCAAG	34	0
ATP6V1C1	CGCTGTTGTTCCAGAGTGGG	39	0
ATP6V1C2	GCCCGTATGCAAATATCCTG	63	0
ATP6V1C2	CCCGCAGGATATTTGCATAC	257	0
ATP6V1C2	TGCAAATATCCTGCGGGGCC	80	0
ATP6V1C2	ATGCAAATATCCTGCGGGGC	212	0
ATP6V1C2	CCGTATGCAAATATCCTGCG	49	0
ATP6V1C2	CCAGAGCCCGGGATAAAAGG	0	0
ATP6V1C2	CCCCGCAGGATATTTGCATA	80	0
ATP6V1C2	AGAGCCCGGGATAAAAGGCG	6	0

ATP6V1C2	CCCGTATGCAAATATCCTGC	41	0
ATP6V1C2	CGGGAAAAGGCTCTGAGCTC	225	0
ATP6V1D	CTTTCTCCACCTTTCGTCTT	82	0
ATP6V1D	CCTTCGTCGCCTGTGACAAC	86	0
ATP6V1D	TGACCCCTCTTGTTGCTAAG	226	0
ATP6V1D	GGAATTGAGGGGTGGTTCCG	86	0
ATP6V1D	GCTGCCTCTTAGCAACAAGA	124	0
ATP6V1D	CACGCTTCGCTCCTAGTCTT	135	0
ATP6V1D	CTGTGACCAAAGACGAAAGG	10	0
ATP6V1D	CGGTTGTCACAGGCGACGAA	74	0
ATP6V1D	TCAAGGCGCGAGAAAGACAT	0	0
ATP6V1D	TCTTGTTGCTAAGAGGCAGC	0	0
ATP6V1E1	TCACGAGTCAGCGACGTAGA	88	0
ATP6V1E1	AGGGGTACATGATTGTTGGC	120	0
ATP6V1E1	CCTACCCAAGATCCCAGTTT	71	0
ATP6V1E1	GCTTAGGGGTACATGATTGT	152	0
ATP6V1E1	TTGACCTCAAGTTCGAGACC	81	0
ATP6V1E1	TCGCTGACTCGTGACCTGAC	43	0
ATP6V1E1	AAACTGGGATCTTGGGTAGG	97	0
ATP6V1E1	GCCCGACTAATTTCAGATAG	282	0
ATP6V1E1	TACACAGGGTTATGGGCTTA	77	0
ATP6V1E1	CCTAAACTGGGATCTTGGGT	67	0
ATP6V1E2	AGAGGATGGCTCTGGCCTTC	21	0
ATP6V1E2	TGACTATTGCGTCATCGTGG	27	0
ATP6V1E2	TAGTTCCGTTGTGCACTTAG	131	0
ATP6V1E2	CCACGTCTTCTCCCAGCATC	470	0
ATP6V1E2	GCATGACTATTGCGTCATCG	0	0
ATP6V1E2	TTGTGCACTTAGAGGCCGAG	101	0
ATP6V1E2	CTCGGCCTCTAAGTGCACAA	156	0
ATP6V1E2	GCACTTAGAGGCCGAGAGGA	67	0
ATP6V1E2	GAGAAGACGTGGAGGCCTCT	38	0
ATP6V1E2	TCAAGGAGGAGAGACCAAAA	20	0
ATP6V1F	AGTTACTCATCTCAAGGGCA	94	0
ATP6V1F	TGCTGTTAATGCGCATGTCC	222	0
ATP6V1F	ACGGCGCAGGCGTATTCACA	59	0
ATP6V1F	CATGCGCATTAACAGCAACA	149	0

ATP6V1F	AATGCGCATGTCCCGGAATG	66	0
ATP6V1F	TTCTGGGAATCGTAGTCCCC	0	0
ATP6V1F	GAACGAATGAATGCCCGCGA	58	0
ATP6V1F	GTAACTGCGTCTCTTCTCTG	0	0
ATP6V1F	TAAAGACAGGCCCGAACCAG	74	0
ATP6V1F	GCGCAGGCGTATTCACATGG	0	0
ATP6V1G1	GGTAGCCATTAAAAGAGGCC	17	0
ATP6V1G1	CTACAGAGTGAACCAGCGAC	0	0
ATP6V1G1	TACAGAGTGAACCAGCGACA	43	0
ATP6V1G1	TCTGTGATTCGGCAGAAATG	178	0
ATP6V1G1	GCTCAACGTCAAGAGAGGAG	0	0
ATP6V1G1	TCAGCTGATGCTACGCTGTG	31	0
ATP6V1G1	CGCTCCCTGGCCTCTTTTAA	110	0
ATP6V1G1	CTTCTGTGATTCGGCAGAAA	102	0
ATP6V1G1	GCTGTGCGGTCACGTGACAA	87	0
ATP6V1G1	GAGCAAGCGCAGATTGTGGG	0	0
ATP6V1G2	AAAGGTCTAGGGATGAGGCA	88	0
ATP6V1G2	CATCCCTAGACCTTTCCGAC	68	0
ATP6V1G2	TGTCATCATGCAACCTGTGT	51	0
ATP6V1G2	ATCCCTAGACCTTTCCGACT	227	0
ATP6V1G2	GAGGACCAGTCATCAATAGG	32	0
ATP6V1G2	GTTAGCCATCCCAGTCGGAA	58	0
ATP6V1G2	GTGAATGCGGTGAAAAGGTA	0	0
ATP6V1G2	CTTGCTCAGGCAGTACCCAA	325	0
ATP6V1G2	CTCATCCTCCTATTGATGAC	86	0
ATP6V1G2	CTAGACCTTTCCGACTGGGA	0	0
ATP6V1G3	TTCCACCTTGGGCTTTCCTT	164	0
ATP6V1G3	GACCTGAAACAAAGTACTGC	51	0
ATP6V1G3	CTTTATTGGGCTGGATAGCT	197	0
ATP6V1G3	GCTTTATTGGGCTGGATAGC	101	0
ATP6V1G3	GTCCCAAGGAAAGCCCAAGG	0	0
ATP6V1G3	TGGGTCCCAAGGAAAGCCCA	0	0
ATP6V1G3	ATTCCACCTTGGGCTTTCCT	82	0
ATP6V1G3	TCAAACTGGCTTTATTGGGC	69	0
ATP6V1G3	CTCTTCTGTATGAGATAAGG	71	0
ATP6V1G3	TCATACAGAAGAGCTAAGAT	27	0

ATP6V1H	TGGGCTCCAGATGGGACTGT	26	0
ATP6V1H	TAAGGCAAAGACAGTTGCTG	17	0
ATP6V1H	AGTTGCTGAGGCCTGGACTA	130	0
ATP6V1H	GGAAAATTTGGGCTCCAGAT	0	0
ATP6V1H	GGGAAAATTTGGGCTCCAGA	0	0
ATP6V1H	GTTGCTGAGGCCTGGACTAG	40	0
ATP6V1H	CCCAAATTTTCCCCTAGTCC	33	0
ATP6V1H	GTAAGAATGTACTGCCCTGG	58	0
ATP6V1H	GCCTGGACTAGGGGAAAATT	0	0
ATP6V1H	GAATAAGAACGCCTGCTTCA	104	0
ATP7A	AAGTGGGGGTAGTCGAGGGA	58	0
ATP7A	CTGGTTTCGCTTTTGTCGTG	320	0
ATP7A	GAGCGACTGGTCACGTGAAA	86	0
ATP7A	CCCTAACAAAGACCCACTCG	79	0
ATP7A	TTACATCTTGGCCTGAGGTT	123	0
ATP7A	TTCGCTTTTGTCGTGGGGGC	1	0
ATP7A	TCTGGTTTCGCTTTTGTCGT	95	0
ATP7A	AGTCGCTCTCTTTCCCGTGG	334	0
ATP7A	CCTCGAGTGGGTCTTTGTTA	302	0
ATP7A	TTCTGGTTTCGCTTTTGTCG	118	0
ATP7B	GCGCTGCAGCAGACGATGAT	13	0
ATP7B	TGAGCTAGCCTCCAGCTTTG	199	0
ATP7B	CTGAGCTAGCCTCCAGCTTT	36	0
ATP7B	CAGGATGACTGGACATATCC	120	0
ATP7B	TGACGTCCGACAAGCCATCT	162	0
ATP7B	CCTGGCTGACATAGCTGACC	31	0
ATP7B	GAGATGGCTTGTCGGACGTC	108	0
ATP7B	CCACTCACCACAAGCTGAGC	115	0
ATP7B	TGGCTGTCCTGCTCAGCTTG	358	0
ATP7B	CGCTGCAGCAGACGATGATG	66	0
ATP8A1	GGTTGGGTTAGACAGTTTCA	58	0
ATP8A1	ACTGTCTAACCCAACCCCTA	67	0
ATP8A1	GGGTTGGGTTAGACAGTTTC	0	0
ATP8A1	CTAACCCAACCCCTAAGGTA	63	0
ATP8A1	TGTTCCTTACCTTAGGGGTT	169	0
ATP8A1	CTGTTCCTTACCTTAGGGGT	103	0

ATP8A1	AGTTAAAAACAGCCTGGGCA	60	0
ATP8A1	CTTTGGGAGGCCAAGGCTGG	236	0
ATP8A1	AGAATCTGTTCCTTACCTTA	333	0
ATP8A1	AAAGTCAGTTAAAAACAGCC	31	0
ATP8A2	GGGCTTTTAGGTGTCGGGGA	35	0
ATP8A2	AGATTGGGCCTGTACTTTCG	83	0
ATP8A2	GTACTTTCGGGGGCTCTTCC	34	0
ATP8A2	CAGGTAAATAGGTAGCAGAC	0	0
ATP8A2	TCCGCAATATTACGCAGGAC	68	0
ATP8A2	GAGATTGGGCCTGTACTTTC	181	0
ATP8A2	CCCGGTCCTGCGTAATATTG	35	0
ATP8A2	CACTTGTCCTGTGCAAGTGG	42	0
ATP8A2	GATTGGGCCTGTACTTTCGG	75	0
ATP8A2	TCCCCGACACCTAAAAGCCC	0	0
ATP8B1	GGGGCATTGTAGCAGCATGC	27	0
ATP8B1	TATGACTCTTGCCCAGTTTT	192	0
ATP8B1	GCAAGAGTCATAATTCACTC	82	0
ATP8B1	TCATGTCAAGTTGAATTTGG	257	0
ATP8B1	GCAGTATTCAACCAAAAACT	0	0
ATP8B1	GGCAGTATTCAACCAAAAAC	0	0
ATP8B1	GTTTCTCTTTCCTTTTAAGC	0	0
ATP8B1	ATTGGAACTACCTGCTTAAA	169	0
ATP8B1	TTCAGTACTGATTAAATGCT	65	0
ATP8B1	TCTTCATGTCAAGTTGAATT	223	0
ATP8B2	TGCGGATGGACGTACGTGTG	110	0
ATP8B2	CGGCAGGAGAGCTGTAGAAG	86	0
ATP8B2	ATGCGGATGGACGTACGTGT	259	0
ATP8B2	GTCTTAGCCCTTCCGAGCCT	163	0
ATP8B2	CAAAACGAGCATCCTCTGCA	27	0
ATP8B2	GAGGCTAGATCGCTCTGAGG	43	0
ATP8B2	GATGCGGATGGACGTACGTG	0	0
ATP8B2	CCTGGCGCCAGGGAAAATTC	71	0
ATP8B2	CCAGAATTTTCCCTGGCGCC	106	0
ATP8B2	GCAAAACGAGCATCCTCTGC	75	0
ATP8B3	AGAAGGAAGGCGAGGACGAG	142	0
ATP8B3	GCAAAGTCAGAGTAGGGGCG	28	0

ATP8B3	TAGTCCCTTCCCCAAAGTCC	195	0
ATP8B3	AGGCCGGGATAGAAGACGCT	60	0
ATP8B3	CTCCCGAGCGTCTTCTATCC	52	0
ATP8B3	CGCTGAGCAAAGTCAGAGTA	87	0
ATP8B3	AAGGGACTAGAAGGAAGGCG	0	0
ATP8B3	AACTGGCTCCACCTTCGCCT	298	0
ATP8B3	TCGCTGAGCAAAGTCAGAGT	50	0
ATP8B3	AGCAAAGTCAGAGTAGGGGC	35	0
ATP8B4	AGCACATCATGTGCAGCCAA	18	0
ATP8B4	ATTGGCTGCACATGATGTGC	0	0
ATP8B4	GCACATCATGTGCAGCCAAT	24	0
ATP8B4	GACCTCTCATGAATCTCTCT	89	0
ATP8B4	CTCCAAGAGAGATTCATGAG	134	0
ATP8B4	ATCCCAGCCTCCTTCTCACC	321	0
ATP8B4	AATTAACTTCTGGGGCCCAT	63	0
ATP8B4	GCTAGGCTCAAACCAAGCCC	0	0
ATP8B4	ATTTGGGATTCCAGGTGAGA	100	0
ATP8B4	GCCCCAGAAGTTAATTGTCA	11	0
ATP9A	GATGTTGTCCGTCATGTCGG	195	0
ATP9A	CGGGATGTTGTCCGTCATGT	113	0
ATP9A	GACGGACACGGGCGCACCAT	39	0
ATP9A	CATGGTGCGCCCGTGTCCGT	310	0
ATP9A	TTCTGGCGCACCGGCTGCAG	149	0
ATP9A	GGACAACATCCCGCTGCAGC	87	0
ATP9A	CAGCGCGGAGCTCGGGCCCA	24	0
ATP9A	CGCTGCAGTGTCCCCGCCCC	282	0
ATP9A	TGCGCCCGTGTCCGTCGGTC	150	0
ATP9A	CGCGGCCCGACCGACGGACA	218	0
ATP10A	GCGCGCGTTTGCAGCGTTTT	33	0
ATP10A	CCGAGCTGGAAACTTCAGAG	0	0
ATP10A	TTGTTTCCCGCTATCCGGCG	56	0
ATP10A	TGGGACCGAACTGATGCTTC	40	0
ATP10A	GAAACAAAAAAAGCCCGAGC	91	0
ATP10A	AAACCGCGAAGCGCGAGAAA	90	0
ATP10A	GGGACCGAACTGATGCTTCT	92	0
ATP10A	AGCGCGCGTTTGCAGCGTTT	159	0

ATP10A	GGTTTCCTCTCTCCTACTGC	139	0
ATP10A	GTTTCCCAGAAGCATCAGTT	53	0
ATP10B	GAACACTGCCTTCACTGTTC	107	0
ATP10B	GAAGGCAGTGTTCTTCGACT	70	0
ATP10B	TTGCTGGCTCATTGATTCAC	97	0
ATP10B	GCCGAGTCCCAGAACAGTGA	224	0
ATP10B	AACCTTGCAGTTCTCTTTGG	81	0
ATP10B	GATTCAGCTGCTGGGTTTCA	0	0
ATP10B	GCCTTCACTGTTCTGGGACT	251	0
ATP10B	GCCCCCCAAAGAGAACTGCA	58	0
ATP10B	AACACTGCCTTCACTGTTCT	138	0
ATP10B	ATTCAGCTGCTGGGTTTCAA	62	0
ATP10D	TCGGTCAAACAGCTGGAGCT	75	0
ATP10D	ACCGCGAGCAACCTGTACTT	130	0
ATP10D	TGTAAGGGCTCTGGGTCACT	114	0
ATP10D	ATTGGCGGTGCACTGCAGCT	70	0
ATP10D	AGGTGAGATTGTAAGGGCTC	15	0
ATP10D	GCGAGCAACCTGTACTTGGG	26	0
ATP10D	CTCGGTCAAACAGCTGGAGC	41	0
ATP10D	GGTGAGATTGTAAGGGCTCT	109	0
ATP10D	TACAGGTTGCTCGCGGTTGA	348	0
ATP10D	AACCGCGAGCAACCTGTACT	164	0
ATP11A	GTCGGCAGCTGCTCCACTGG	61	0
ATP11A	GGCAGGGGAGGAGGAGACTC	57	0
ATP11A	ACTCGGGAGGAGCAGAGCGC	33	0
ATP11A	GGCGCCTTCTCTCTCCCCGT	32	0
ATP11A	CTGGCGGCAAGGCTGCTGCT	127	0
ATP11A	CAGCTGCTCCACTGGCGGCA	184	0
ATP11A	TCCAGCCGAGCCCAACCGAG	106	0
ATP11A	TGGCGGCAAGGCTGCTGCTC	0	0
ATP11A	AGGGGAGGAGGAGACTCGGG	0	0
ATP11A	AGCAGCAGCCTTGCCGCCAG	26	0
ATP11B	CATCTCCGGAGTCTGCAGCT	0	0
ATP11B	AGAAACAGCGTTCGAGAACC	0	0
ATP11B	GCAACAGGAGGTAGAGACCG	0	0
ATP11B	TTAAGTCGTGTCTGTGGGTC	112	0

ATP11B	CAGGTCTTTTTCGGGCTCTG	86	0
ATP11B	TGGGATTAAGTCGTGTCTGT	57	0
ATP11B	TTAATCCCACTCCCAGAACG	0	0
ATP11B	AAGCGAGCGTGGAAAGGTGC	0	0
ATP11B	GTGGGATTAAGTCGTGTCTG	79	0
ATP11B	GCCCGAAAAAGACCTGCAAC	42	0
ATP11C	TTCAACCCTCTGGGCAAACC	286	0
ATP11C	CGAATCCTGGGAGCTTTCCC	28	0
ATP11C	ACGGTTGGACTACAACTCCC	42	0
ATP11C	TCGGTAGCCTTCCTAGCGGT	81	0
ATP11C	CGCAAATTCCAGCTCTAAGC	0	0
ATP11C	AAATTCCAGCTCTAAGCTGG	116	0
ATP11C	TAGGCAAAGCTCCGAATCCT	117	0
ATP11C	TGCGCACATTTTGCGTTCCA	208	0
ATP11C	CAAACCCGGGACTAGCGTGT	30	0
ATP11C	AGGGTTGAAGAAGGAAGCGC	0	0
ATP12A	GTCGGGAGAGTCCAGGATTG	242	0
ATP12A	TTTAGTAGCCGAACGCCAGG	27	0
ATP12A	TAAAGGGAAGGGGATTCCCA	76	0
ATP12A	CCCTGGCGTTCGGCTACTAA	145	0
ATP12A	CCTGGCGTTCGGCTACTAAA	90	0
ATP12A	CCCTTTAGTAGCCGAACGCC	34	0
ATP12A	CCTTTAGTAGCCGAACGCCA	210	0
ATP12A	CGTTCGGCTACTAAAGGGAA	36	0
ATP12A	AGTACAGCCCTGGTTCCGCT	187	0
ATP12A	CTAAAGGGAAGGGGATTCCC	105	0
ATP13A1	TTAGGGTGTCAGGTGAAATG	164	0
ATP13A1	TCCACGGAGGGGAAGATTGC	177	0
ATP13A1	TGAGGTCTCCTTAGGGTGTC	53	0
ATP13A1	CTTAGGGTGTCAGGTGAAAT	135	0
ATP13A1	AGTCTGGGAACTGTCCTCAC	121	0
ATP13A1	CCTTAGGGTGTCAGGTGAAA	76	0
ATP13A1	CCATTTCACCTGACACCCTA	306	0
ATP13A1	AAGTCTGGGAACTGTCCTCA	0	0
ATP13A1	TCTTGGCCAATGCCCCTGAG	62	0
ATP13A1	TCAGGTGAAATGGGGAAGTC	47	0

ATP13A2	GACCGGAGACACACACCTCA	147	0
ATP13A2	GCCTGGAAGACATGGAGAGC	0	0
ATP13A2	CACGCACAGTCCCTAACCAC	219	0
ATP13A2	TGGTTAGGGACTGTGCGTGC	53	0
ATP13A2	CCAGCACGTCCCAGTGGTTA	176	0
ATP13A2	ACGCACAGTCCCTAACCACT	1	0
ATP13A2	TAACCACTGGGACGTGCTGG	113	0
ATP13A2	CTCCGTGAGGTGTGTGTCTC	27	0
ATP13A2	TCCAGCTCTCCATGTCTTCC	113	0
ATP13A2	GCCTGCTCCTCCGCACACAC	140	0
ATP13A4	GTAGTTGGACCACTTTGCCA	200	0
ATP13A4	GGCAGGGTGCAGTGTTGACT	129	0
ATP13A4	CAGCGTCATCTCAAGCTCCA	58	0
ATP13A4	TCCACAGGAAATGAGTGCCT	77	0
ATP13A4	AAGGCACTCATTTCCTGTGG	226	0
ATP13A4	GCGTCATCTCAAGCTCCAAG	61	0
ATP13A4	TTGAACCTGTGCACTGCCTC	129	0
ATP13A4	GCAACGTACAGCTTCCCCTC	77	0
ATP13A4	TGCCAAGGCATTGTTTCTTC	100	0
ATP13A4	CACTGCCTCTGGTAATGCAG	63	0
ATP13A5	GGAATGACGGGGGAAGAAGT	56	0
ATP13A5	CAAGACCTGAGGAATGACGG	0	0
ATP13A5	CCTCAAGACCTGAGGAATGA	101	0
ATP13A5	CCGTCATTCCTCAGGTCTTG	246	0
ATP13A5	GGGGTGACAATGAGAATTGA	0	0
ATP13A5	TATGAGTGCCTCAAGACCTG	30	0
ATP13A5	TTCTTCCCCCGTCATTCCTC	40	0
ATP13A5	TCAAGACCTGAGGAATGACG	126	0
ATP13A5	CTCAAGACCTGAGGAATGAC	45	0
ATP13A5	AGAATTGAAGGAAGGGAGGT	0	0
SV2A	TGAGCATCGGGAGAGACCAT	113	0
SV2A	ATACTGAGACTGGGGTGCTA	68	0
SV2A	TTACTAGGAAGGGCCTGGGA	0	0
SV2A	GCAGGACCCAATAATTTCAG	262	0
SV2A	GGTCCTGCCATACTGAGACT	29	0
SV2A	GCATCGGGAGAGACCATTGG	0	0

SV2A	GCTGTTCTTGTGACAACCTC	36	0
SV2A	TACTAGGAAGGGCCTGGGAA	29	0
SV2A	GAATAGGAGCACTCTGGATT	166	0
SV2A	CACGGGGAGAAATGATGAAA	0	0
NPC1L1	ACCCTGGCCTGACTGGGTTA	141	0
NPC1L1	ACCCTAGCACCTGCGTGATG	92	0
NPC1L1	CCTCTGGCTTCGGTCGCTTT	131	0
NPC1L1	ACTGTTTGGCCCCAGTCCAC	85	0
NPC1L1	ATGACAACCCTGGCCTGACT	45	0
NPC1L1	AGGCCAGGGTTGTCATCGAA	104	0
NPC1L1	ATGAGGCTCATCTAAAGCGG	49	0
NPC1L1	TTAGATGAGCCTCATCACGC	75	0
NPC1L1	TCATCTAAAGCGGGGGCCTG	62	0
NPC1L1	CCAAAGCGACCGAAGCCAGA	37	0
UCP1	TATATAGCTGGGCCGCTCCC	158	0
UCP1	CTGTTTTTTGAACCGACCGC	129	0
UCP1	CCGCTGGGACTTATATAGCT	35	0
UCP1	GCACCGAGCGAGAATGGGAA	46	0
UCP1	TCCGCTGGGACTTATATAGC	36	0
UCP1	TGTTTTTTGAACCGACCGCC	154	0
UCP1	CCCAGCTATATAAGTCCCAG	31	0
UCP1	TATAAGTCCCAGCGGAAGAC	118	0
UCP1	AAATCGGGTTTGCTGCCCGG	122	0
UCP1	CAGAAATCGGGTTTGCTGCC	133	0
UCP2	TGTCCTGGTCCCTAAACGCC	0	0
UCP2	CAACGGCTGTTGTCGGTTGC	75	0
UCP2	GCTGTAACCAATCGACAGCG	52	0
UCP2	CTGACGCCTTTTCTCATCCC	83	0
UCP2	TAATTGGCTGACCCGTCCTG	467	0
UCP2	AATTAAAGGCGAACCAGGCC	74	0
UCP2	CCAATCAGAACTGCAGCAAC	278	0
UCP2	CTGGGATGAGAAAAGGCGTC	0	0
UCP2	AAGGCGTCAGGAGATGGACC	51	0
UCP2	GGCAACCGACAACAGCCGTT	97	0
UCP3	GGAGTTTTTCCAGGGTGAGA	1	0
UCP3	TGGAGTTTTTCCAGGGTGAG	35	0

UCP3	TTTTCATCACCCTCTCACCC	515	0
UCP3	TGGAAAAACTCCATACACAT	36	0
UCP3	TTTCCACAATCCTATGAGAT	53	0
UCP3	AATGTGTATGGAGTTTTTCC	79	0
UCP3	CCTAATAGTACCTATCTCAT	42	0
UCP3	CCTATGAGATAGGTACTATT	40	0
UCP3	ATGTGTATGGAGTTTTTCCA	115	0
UCP3	GTACCTATCTCATAGGATTG	46	0
RHAG	AGGCCTGTGCAATAGATTGT	24	0
RHAG	GCATAATTTTCCCCAATGGG	74	0
RHAG	TTAAAATGCCCCACCCATTG	271	0
RHAG	GATTAAAATGCCCCACCCAT	54	0
RHAG	ACAAAGGACTGCTCTGTGTG	232	0
RHAG	CAGAGCATAATTTTCCCCAA	66	0
RHAG	ATAGATTGTTGGCATCATCC	1	0
RHAG	GCAATGAAATTGTGAGAACC	158	0
RHAG	ATGCCAACAATCTATTGCAC	82	0
RHAG	ATTGTGGTAGGCTTTGTAGA	131	0
RHBG	CGCTGATCCTGGCACTGTTA	143	0
RHBG	CCCACAGCTGTGCCCTATCA	78	0
RHBG	TAAATGGCCCTTAAAGACCC	7	0
RHBG	CAAGGTAGGGCCAAGACTTG	0	0
RHBG	CCCTATCAGGGCAGAACACA	0	0
RHBG	TAAAGACCCCGGTCTGGGCT	55	0
RHBG	CTGTTACGGTGCCAGGTCCT	0	0
RHBG	GCCCAGACCGGGGTCTTTAA	31	0
RHBG	GCCCTTAAAGACCCCGGTCT	38	0
RHBG	TCCCACAGCTGTGCCCTATC	197	0
RHCG	TGTGTGTCCCGTTGATACTG	0	0
RHCG	AGAAAGGCCCCAGTATCAAC	86	0
RHCG	CCGACGCGGGGAATCTTTTG	1	0
RHCG	TCTGTGTGTCCCGTTGATAC	200	0
RHCG	TCTTTTGGGGCCCTCGGCTT	0	0
RHCG	CTGTGTGTCCCGTTGATACT	153	0
RHCG	GAGAAAGGCCCCAGTATCAA	0	0
RHCG	CCCAAAAGATTCCCCGCGTC	52	0

RHCG	AACGGGACACACAGAATTTG	118	0
RHCG	CCCCAAAAGATTCCCCGCGT	71	0
MTCH1	GCTGCCAGACGTCCCCAATA	117	0
MTCH1	CAGACCATATTGGGGACGTC	72	0
MTCH1	GGAAGCGCCTCAGACCATAT	64	0
MTCH1	CGAGCTGAATCTCCTGCTGC	135	0
MTCH1	GAAGCGCCTCAGACCATATT	112	0
MTCH1	GACGTCCCCAATATGGTCTG	343	0
MTCH1	GAAAACTCCCGAAGCCAGGG	0	0
MTCH1	ATGCGATCGGACAAAGAGGC	37	0
MTCH1	CAGAGAAAACTCCCGAAGCC	23	0
MTCH1	TGCGATCGGACAAAGAGGCT	123	0
MTCH2	CTTCTGTTTCCGGACACTTG	36	0
MTCH2	GCAAGAGAAACCGTCATTCC	22	0
MTCH2	TCGCCCTTCACCAGGAATGA	215	0
MTCH2	GAAACCGTCATTCCTGGTGA	77	0
MTCH2	TCTCTTGCACATGCGCTTCC	79	0
MTCH2	AAACCGTCATTCCTGGTGAA	32	0
MTCH2	AAGAATGGGAGTGTCAGTGG	35	0
MTCH2	TGACCTTGCCTCTGCCGTCA	232	0
MTCH2	CAGAAGAATGGGAGTGTCAG	0	0
MTCH2	AGAAGAATGGGAGTGTCAGT	1	0
FLVCR2	AATATGAGGGGCAGTGCAAA	46	0
FLVCR2	GAAGGCAAGAGACACTGGGT	0	0
FLVCR2	CGGCGCCACCTAAGTGTTCT	193	0
FLVCR2	AGTGTTCTGGGGAATTCAAG	38	0
FLVCR2	CCAGAACACTTAGGTGGCGC	88	0
FLVCR2	GGGAAGGCGGATCTTCTTGC	30	0
FLVCR2	GAATATGAGGGGCAGTGCAA	134	0
FLVCR2	CTTGCAGGGTCCGCTGATAG	269	0
FLVCR2	GGAATGCATCCCGCAAGCTG	106	0
FLVCR2	TGGGCATTACTTCGGGAAGG	0	0
MFSD7	CTGTGTGTTGAGCCCTATCA	35	0
MFSD7	TGTGTGTTGAGCCCTATCAT	127	0
MFSD7	CCTATCATGGGCTGTGCAGC	18	0
MFSD7	GCATGTGTATCAGGTGCGGG	120	0

MFSD7	GCGGGTGGTGTGTTTGTGCT	77	0
MFSD7	CGTGCATGTGTATCAGGTGC	46	0
MFSD7	CACACAATACACATGGGCAC	331	0
MFSD7	CGGGTGGTGTGTTTGTGCTT	143	0
MFSD7	CTCAACACACAGACACAGGT	70	0
MFSD7	CAGCTGCACAGCCCATGATA	52	0
DIRC2	TACCCATTTCGCCTGCCAAT	189	0
DIRC2	TCACTGGAGATGGATCTTTG	169	0
DIRC2	CTTTCTTCTCGTTTACCCTG	0	0
DIRC2	GTTTAAGCGTAGTTGGTCCC	57	0
DIRC2	TTAAGCGTAGTTGGTCCCAG	105	0
DIRC2	TCGCCTATTGGCAGGCGAAA	121	0
DIRC2	TTTATTGCTCGCCTATTGGC	0	0
DIRC2	CGCCTATTGGCAGGCGAAAT	0	0
DIRC2	TTTAAGCGTAGTTGGTCCCA	43	0
DIRC2	TGCCAATAGGCGAGCAATAA	127	0
ATP13A3	GATGGAGTCCAAACAGCTTG	106	0
ATP13A3	TACAGGTAATCTCATGGTGG	117	0
ATP13A3	CTGCAGAACCTCAAGCTGTT	0	0
ATP13A3	TCACCTCGGGACTTCCACAC	147	0
ATP13A3	CTCATGGTGGAGGTGTATTC	80	0
ATP13A3	CAGAGATGCCTCACTGTGAT	44	0
ATP13A3	CTGTTTGGACTCCATCTTCT	321	0
ATP13A3	AGTGAGGCATCTCTGCTCTG	98	0
ATP13A3	ACTGATTGCCAGCTCCAGTG	129	0
ATP13A3	GTGTGGAAGTCCCGAGGTGA	80	0
SLN	TGCAGTCTTGCATAACAGGT	97	0
SLN	GCTCATGAACAAGTACTGTC	84	0
SLN	AGGCGTCACCTCACCAATCC	43	0
SLN	GGTATGCAGTCTTGCATAAC	62	0
SLN	ACCCATCTTAAGTCCTGGAT	197	0
SLN	TCTTAAGTCCTGGATTGGTG	104	0
SLN	ATGCAGTCTTGCATAACAGG	32	0
SLN	TGAGCCACCCCCAAAGTTGC	0	0
SLN	CACCAATCCAGGACTTAAGA	158	0
SLN	ATTTCCAGCAACTTTGGGGG	83	0

ASNA1	ATAGGTCCCCGGGCTGATAG	25	0
ASNA1	ACTCTAAGCCACTATCAGCC	88	0
ASNA1	GGACCTACCGTGAAGAATAG	78	0
ASNA1	CTAACTTCACCGAAAGGGCA	66	0
ASNA1	TCTAAGCCACTATCAGCCCG	1	0
ASNA1	GACCTACCGTGAAGAATAGA	89	0
ASNA1	GTAGGTCCCAACCTCACCTC	205	0
ASNA1	TCACCGAAAGGGCATGGACT	114	0
ASNA1	TTCACCGAAAGGGCATGGAC	125	0
ASNA1	CGCCCCCTCTGTTATTCAAT	120	0
MMGT1	GGCCTACGGGAGGATGGGTG	103	0
MMGT1	CCACTCGACCGCACCCACCA	74	0
MMGT1	CACGAGGGTGGGTGTAGCCG	109	0
MMGT1	CGAGGGTGGGTGTAGCCGAG	0	0
MMGT1	GGAGGATGGGTGGGGCGTAG	69	0
MMGT1	ACGAGGGTGGGTGTAGCCGA	121	0
MMGT1	TGGTGGGTGCGGTCGAGTGG	161	0
MMGT1	GAGAGTGGGTGAGGGCCGAG	33	0
MMGT1	GAGGGGACGGAGAGTGGGTG	81	0
MMGT1	CGGCTACACCCACCCTCGTG	144	0
MAGT1	CTTTTTTCTATTGGCCGACC	138	0
MAGT1	TTTACGTATCGAGAAAGGGG	20	0
MAGT1	AAATGGGACCTAGCCGCGTG	112	0
MAGT1	TTACGTATCGAGAAAGGGGC	0	0
MAGT1	AATAGCTCTTATCCCTCACG	86	0
MAGT1	CATGAAGGCGGCGAATTTCA	29	0
MAGT1	CACAACTGCGCATGCGTGCT	0	0
MAGT1	CTCTTATCCCTCACGCGGCT	203	0
MAGT1	TTTTTTCTATTGGCCGACCG	33	0
MAGT1	AATGGGACCTAGCCGCGTGA	301	0
MFSD1	CGTTCCTTCCAGCATTTATG	147	0
MFSD1	TGCAGACGCTGTGCACCGAA	21	0
MFSD1	GCACAGCGTCTGCAGAAATC	49	0
MFSD1	GCTTTGGGAAATTGAGGAAC	52	0
MFSD1	CACGCCCTGGGATACTTCAG	145	0
MFSD1	TCTTGACTGCTCCCACACCT	130	0

MFSD1	CCACGCCCTGGGATACTTCA	211	0
MFSD1	AAGCATCTCTTTCCCAGGTG	57	0
MFSD1	CTCTTGACTGCTCCCACACC	186	0
MFSD1	GAAGTATCCCAGGGCGTGGA	19	0
MFSD10	AGACCAGGGTGAGCACATCG	105	0
MFSD10	GCAACGGTCCTGAGAGACCA	34	0
MFSD10	GAGAGAAAACTTGAAGCCGC	0	0
MFSD10	CAGCAGGAAACGCAGGCTTC	72	0
MFSD10	AGTTTTCTCTCGCTGTTACG	121	0
MFSD10	AGAGAAAACTTGAAGCCGCG	155	0
MFSD10	CGTTTCCTGCTGCCTGGGTT	63	0
MFSD10	CGAGAGAAAACTTGAAGCCG	0	0
MFSD10	GAGCACCCTAAAGGGGCTGA	0	0
MFSD10	CCCTAAAGGGGCTGATGGCA	153	0
MFSD11	CCACAGAAACTGGTCCCTGG	105	0
MFSD11	TACAGGTATTGCACTGGGTG	23	0
MFSD11	GTATTGCACTGGGTGGGGAA	49	0
MFSD11	CCTCCAGGGACCAGTTTCTG	81	0
MFSD11	CAGTTTCTGTGGGCGTGTAT	0	0
MFSD11	ACTGATTTGGCTGATCCTCC	151	0
MFSD11	TTACAGGTATTGCACTGGGT	41	0
MFSD11	CTGTGGGCGTGTATTGGAGC	56	0
MFSD11	GTTACAGGTATTGCACTGGG	174	0
MFSD11	CAATACACGCCCACAGAAAC	36	0
MFSD12	ATCTGCTCACCTTGGGCAGG	209	0
MFSD12	TGCCCAAGGTGAGCAGATTG	55	0
MFSD12	ATTGGTAGTGACTGCCCAGT	29	0
MFSD12	CAGGCATGCCGTGTCAGTCA	71	0
MFSD12	ATGTACAGTATGCCCACCTG	53	0
MFSD12	TGTACAGTATGCCCACCTGT	116	0
MFSD12	TTGGTAGTGACTGCCCAGTG	187	0
MFSD12	GAGCGAGTAGGTGAGGTACA	148	0
MFSD12	CATTGGTAGTGACTGCCCAG	105	0
MFSD12	TTGATGAGTGACGTTTGCCC	80	0
MFSD2A	GGGCTGGGCAGAACTATAAG	55	0
MFSD2A	GGCAGATCACTTTCAGCCTG	45	0

MFSD2A	ACCCATGACTTCTGAGCCCC	132	0
MFSD2A	GGCTGAAAGTGATCTGCCCG	18	0
MFSD2A	GCTCAGAAGTCATGGGTGGT	27	0
MFSD2A	CTCCTAGCAATCCGAGAAGC	40	0
MFSD2A	CCCAGGCCTAGGAAAGGTAG	29	0
MFSD2A	TCAGAGCCCCTACCTTTCCT	78	0
MFSD2A	GCCTGGGGCTCAGAAGTCAT	191	0
MFSD2A	ATAGTTCTGCCCAGCCCTCT	158	0
MFSD2B	CCTCAGGACCGTTCTCCTTG	167	0
MFSD2B	CAGCGAAGAACCAAAATCCG	44	0
MFSD2B	GCCTCTTGCTTCATTTTGCA	68	0
MFSD2B	GAAGAACCAAAATCCGCGGG	25	0
MFSD2B	CATGGTCCAGCCTGAACGAG	74	0
MFSD2B	GGAAACGCAGAAACCGCGAA	0	0
MFSD2B	CGTTGGGCATCATGTGTAGC	31	0
MFSD2B	ATGGTCCAGCCTGAACGAGC	255	0
MFSD2B	AGCGAAGAACCAAAATCCGC	39	0
MFSD2B	CCCTCAGGACCGTTCTCCTT	180	0
MFSD3	GCGGTATCGCAGAGCATGGT	67	0
MFSD3	CTCTGCGATACCGCAGGTGA	31	0
MFSD3	TCTGCGATACCGCAGGTGAG	37	0
MFSD3	ACCTGCGGTATCGCAGAGCA	48	0
MFSD3	ACCATGCTCTGCGATACCGC	0	0
MFSD3	TGCGGTATCGCAGAGCATGG	39	0
MFSD3	CCGAGAAACTGCTGAGAGTC	5	0
MFSD3	CCGGACTCTCAGCAGTTTCT	35	0
MFSD3	TGCGAGGCCGCAGGTAAAGA	122	0
MFSD3	AAACTGCTGAGAGTCCGGCC	75	0
MFSD5	GGGGAACCACGTGGATATCT	196	0
MFSD5	AATGTCACCTGATCTTGGCT	94	0
MFSD5	AAATGTCACCTGATCTTGGC	232	0
MFSD5	AACAGAGAACTGGAGGAGGT	0	0
MFSD5	TTTGAACAGAGAACTGGAGG	0	0
MFSD5	TCTTCTCCAAGATATCCACG	160	0
MFSD5	ACAGAGAACTGGAGGAGGTG	34	0
MFSD5	AGAGAACTGGAGGAGGTGGG	83	0

MFSD5	GAACAGAGAACTGGAGGAGG	46	0
MFSD5	CAGAGAACTGGAGGAGGTGG	0	0
MFSD6	TACCGGCTGGACCCGTTTCA	387	0
MFSD6	GCTGAGGAGGCGAAAGCACT	39	0
MFSD6	AAGTCGCTTGGGGGTGCGGG	0	0
MFSD6	AGGAGCCGCAGCGGGAGAGA	20	0
MFSD6	CTACCGGCTGGACCCGTTTC	241	0
MFSD6	GTCCAGCCGGTAGTGACCGG	3	0
MFSD6	CTCAGCGCCCGGCCCTGAAA	0	0
MFSD6	GGCTGGACCCGTTTCAGGGC	0	0
MFSD6	ATGCGTCGACTGCCCGCTCC	173	0
MFSD6	GGAGGCGAAAGCACTGGGCG	112	0
MFSD6L	AAACCGTCAGAGTCGGCGTG	148	0
MFSD6L	TGTAGGGTGCGCTCTGAGCT	181	0
MFSD6L	TGGGATTTGCGATGTCAGAG	0	0
MFSD6L	TAAAACCGTCAGAGTCGGCG	0	0
MFSD6L	CATCTTCTTCAGGTCTGTAC	189	0
MFSD6L	TGGAATGAATGGTATCCACC	154	0
MFSD6L	TCTGTGGTTGGTCACTGATC	183	0
MFSD6L	GGCGTTTAGCTCAGGCTCCT	89	0
MFSD6L	GCAGTCCAGGCGTTTAGCTC	35	0
MFSD6L	AAAACCGTCAGAGTCGGCGT	74	0
MFSD8	TGCAAAGGCGCCTTACCTGG	108	0
MFSD8	AGCCACCTCTACCTTTCACG	382	0
MFSD8	TCTACCTTTCACGGGGGGAA	222	0
MFSD8	CCACCTCTACCTTTCACGGG	165	0
MFSD8	GCTTGTAACCTGCAGGTCCC	165	0
MFSD8	CTCTACCTTTCACGGGGGGA	28	0
MFSD8	AACTCCCACGTTTCAGCAGC	129	0
MFSD8	TCAGAGCCAGGACTGAAGGT	99	0
MFSD8	TCGGAGGGCTGAGATAATGG	32	0
MFSD8	GCTTCGGAGGGCTGAGATAA	161	0
MFSD9	GGGCGCTGTGTTAGCCTTAG	34	0
MFSD9	CCATGATGAAAAGGGCACTT	40	0
MFSD9	CTAAACAAGCGACACTCTAG	71	0
MFSD9	AGACTGCCCCACGCCAATAA	243	0

MFSD9	CCAAAGTGCCCTTTTCATCA	162	0
MFSD9	AGCGACACTCTAGTGGTGAT	70	0
MFSD9	AATAAGGGTGCCGCTGGCCT	155	0
MFSD9	ATCATCGCCCCTCTCCTCTC	108	0
MFSD9	CATCATCGCCCCTCTCCTCT	152	0
MFSD9	GGACAACTGCACCCATGGTG	58	0
NIPA1	GGGTGAGTCCAACCACTTAC	193	0
NIPA1	AACCACTTACCGGGTCCCCT	47	0
NIPA1	CAAATCCTAGGTGGAGTTGA	80	0
NIPA1	AAGTACCCTCAACTCCACCT	269	0
NIPA1	TCAAATCCTAGGTGGAGTTG	135	0
NIPA1	CTCCACCTAGGATTTGACCA	122	0
NIPA1	GGTGAGTCCAACCACTTACC	140	0
NIPA1	AGAGCACAAGAGGGCGGACA	40	0
NIPA1	GGACACTGCATTCTGCAGAA	163	0
NIPA1	AGGACACTGCATTCTGCAGA	51	0
NIPA2	CTGGGGCCTTGTAAAGGAAC	76	0
NIPA2	AGCCGACTGGGGCCTTGTAA	171	0
NIPA2	CAGTACAGTATGCTGCCAGG	104	0
NIPA2	TAAAACGAGGATTCCGGGCC	36	0
NIPA2	CGTGGCCCTTATTTAAAACG	46	0
NIPA2	AAGCAGTACAGTATGCTGCC	104	0
NIPA2	GTTTTAAATAAGGGCCACGC	76	0
NIPA2	TAAAGGAACCGGAACCCGGC	0	0
NIPA2	CGGGTTCCGGTTCCTTTACA	170	0
NIPA2	CTTGTAAAGGAACCGGAACC	41	0
NIPAL1	CGATAACAAACTCCCGAACG	39	0
NIPAL1	CACTGCTTTTCCCCTCGTTC	39	0
NIPAL1	GCTGCTCTTTTCGGCACTCT	42	0
NIPAL1	TCGTTCGGGAGTTTGTTATC	103	0
NIPAL1	CTCGTTCGGGAGTTTGTTAT	55	0
NIPAL1	TCACTGCTTTTCCCCTCGTT	233	0
NIPAL1	TGCGGTAGTCTGCAGGGCTT	0	0
NIPAL1	TTTCGGGTGGCTGCTCTTTT	107	0
NIPAL1	CACTTCTCCAGCCTTTCGGG	196	0
NIPAL1	TGACTAGTAACTGCCTTAGG	227	0

NIPAL4	ATCAATGAACGGGTAAGGCA	147	0
NIPAL4	AAGGAACCCAGGTTCCCAAT	31	0
NIPAL4	CATCCCCAGGGAAAGAGTAC	0	0
NIPAL4	GAGGTGGAAGTGGCAACATA	50	0
NIPAL4	CTGCTGCCAATTGGGAACCT	149	0
NIPAL4	GGAAGTGGCAACATAGGGGT	0	0
NIPAL4	CCCGTTCATTGATTCAACCC	3	0
NIPAL4	CGAGGTGGAAGTGGCAACAT	113	0
NIPAL4	TACCAGCACAGATCAGACCT	64	0
NIPAL4	AGGTGGAAGTGGCAACATAG	22	0
ATP5L2	AAGTGAACCACTCAGGGTCC	28	0
ATP5L2	CTTGTATAAGTGAACCACTC	0	0
ATP5L2	TCCTTGTTCTCACGTTGAGA	83	0
ATP5L2	AACCACTCAGGGTCCTGGCC	40	0
ATP5L2	CTGAGTGGTTCACTTATACA	99	0
ATP5L2	GCCTTCTCAACGTGAGAACA	107	0
ATP5L2	TTCTCACGTTGAGAAGGCTG	86	0
ATP5L2	GAACCACTCAGGGTCCTGGC	0	0
ATP5L2	GCTGTCTCGGGTGACAGAAG	41	0
ATP5L2	AAGGCTGAGGAGGAGGCAGG	0	0
